# Genome-wide identification of autosomal genes with allelic imbalance of chromatin state

**DOI:** 10.1371/journal.pone.0182568

**Published:** 2017-08-10

**Authors:** Andrej J. Savol, Peggy I. Wang, Yesu Jeon, David Colognori, Eda Yildirim, Stefan F. Pinter, Bernhard Payer, Jeannie T. Lee, Ruslan I. Sadreyev

**Affiliations:** 1 Department of Molecular Biology, Massachusetts General Hospital, Boston, MA, United States of America; 2 Department of Genetics, Harvard Medical School, Boston, MA, United States of America; 3 Howard Hughes Medical Institute, Boston, MA, United States of America; 4 Department of Pathology, Massachusetts General Hospital and Harvard Medical School, Boston, MA, United States of America; Florida State University, UNITED STATES

## Abstract

In mammals, monoallelic gene expression can result from X-chromosome inactivation, genomic imprinting, and random monoallelic expression (RMAE). Epigenetic regulation of RMAE is not fully understood. Here we analyze allelic imbalance in chromatin state of autosomal genes using ChIP-seq in a clonal cell line. We identify approximately 3.7% of autosomal genes that show significant differences between chromatin states of two alleles. Allelic regulation is represented among several functional gene categories including histones, chromatin modifiers, and multiple early developmental regulators. Most cases of allelic skew are produced by quantitative differences between two allelic chromatic states that belong to the same gross type (active, silent, or bivalent). Combinations of allelic states of different types are possible but less frequent. When different chromatin marks are skewed on the same gene, their skew is coordinated as a result of quantitative relationships between these marks on each individual allele. Finally, combination of allele-specific densities of chromatin marks is a quantitative predictor of allelic skew in gene expression.

## Introduction

Regulation of gene expression involves a plethora of epigenetic mechanisms, with chromatin state [1–5] being a key determinant of gene activity. In particular, combinations of various inter-related covalent histone modifications on promoters and gene bodies have been associated with the level of gene expression [6–11]. Most of these associations have been analyzed in diploid mammalian cells where the levels of chromatin marks, DNA-binding proteins, and expression are a sum of levels from two individual alleles. Multiple efforts to deconstruct these sums into individual allelic levels have identified a number of genes with unequal expression levels of the two alleles. In particular, many genes are subject to genomic imprinting [12–16], X-chromosome inactivation (XCI) [14,17], or random monoallelic expression (RMAE) [16,18–27]. RMAE has been observed in at least two different forms [28]: short-lived stochastic transcription from a single allele in a single cell [27,29–31], and long-term RMAE that can be robustly propagated through cell divisions and differentiation [16,24–26]. Estimates of the fraction of autosomal genes that are subject to RMAE has varied widely, from as low as 0.5% to as high as 25% [28].

Mechanisms mediating RMAE are not fully understood. For the long-term RMAE with stable mitotic transmission, differing epigenetic states of the two alleles has been suggested as a central mechanism, including allelic skews in the levels of histone modifications [32–34] and potentially DNA methylation [14,26]. Many of the existing allele-specific epigenetic surveys have focused on XCI as a model of clear and systematic allelic skew. Direct analyses of allelic skew in epigenetic states of autosomal genes were often limited to a panel of representative genes [24–26] or to genome-wide prediction of monoallelic expression from the composite epigenetic readouts from both alleles [21].

We previously demonstrated that high-resolution allele-specific ChIP-seq is a powerful method for analyzing allelic imbalance of epigenetic states and predicting allele-specific expression on the X-chromosome during XCI [11,33,34]. Here we analyze allelic skew of chromatin state among all autosomal genes across the genome. We perform a quantitative survey of chromatin marks on each allele in order to assess the degree of independence between chromatin states of two alleles, relationships between different marks on the same allele, and relationships between levels of the same mark on two alleles. Finally, we find that the level of allelic skew in a gene’s chromatin state is a quantitative predictor of allelic skew in gene expression.

## Results and discussion

### Allelic skew of chromatin marks is detected in 4% of autosomal genes

High-resolution, allele-specific ChIP-seq was carried out in a clonal F1(*Mus musculus* [mus] x *Mus castaneus* [cas]) female mouse embryonic fibroblast line (33, 34). We surveyed genome-wide profiles for multiple chromatin epitopes, including those associated with transcription activation (phospho-serine-5 RNA polymerase II (POL2S5) and trimethylated histone H3 at lysine 4 [K4me3]), transcription elongation through the gene body (phospho-serine-2 RNA polymerase II [POL2S2] and trimethylated H3 at lysine 36 [K36me3]), and transcription repression (trimethylated H3 at lysine 27 [K27me3]). We also measured corresponding levels of RNA expression using allele-specific RNA-seq. We calculated allelic skew based on the numbers of allele-specific reads, and defined a gene as skewed if it had a sufficient total number of allelically assigned reads, ≥ 2-fold difference between read numbers mapped to maternal and paternal allele, and a high statistical significance of the skew (see SI Methods). With ~22 million SNP and ~1 million insertion/deletion differences between *mus* and *cas* genomes [35], 16674 (93.8%) autosomal genes had at least one allele-specific SNP or indel along the gene body and 16603 (93.4%) had at least one SNP or indel within TSS-proximal region (TSS +/- 3Kbp). Among all reads produced in ChIP-seq experiments, approximately 37% could be assigned to an individual allele (Panels A and B in S1 Fig). Fractions of allele-specific reads in each ChIP-seq experiment are shown in S2 Fig. After filtering by the number of allele-specific reads assigned to a gene, 30–50% of genes contained enough allele-specific reads to reliably calculate an allelic skew (Panel B in S1 Fig). To evaluate the extent of potential large-scale structural genomic variations (aneuploidy, deletions or amplifications of large chromosomal regions etc.) in our MEF line, we compared the numbers of reads assigned to the two alleles on each chromosome. S3 and S4 Figs show a largely balanced representation of the two alleles on individual chromosomes in all ChIP-seq and RNA-seq experiments, which rules out systematic large-scale chromosomal aberrations.

**Fig 1:**
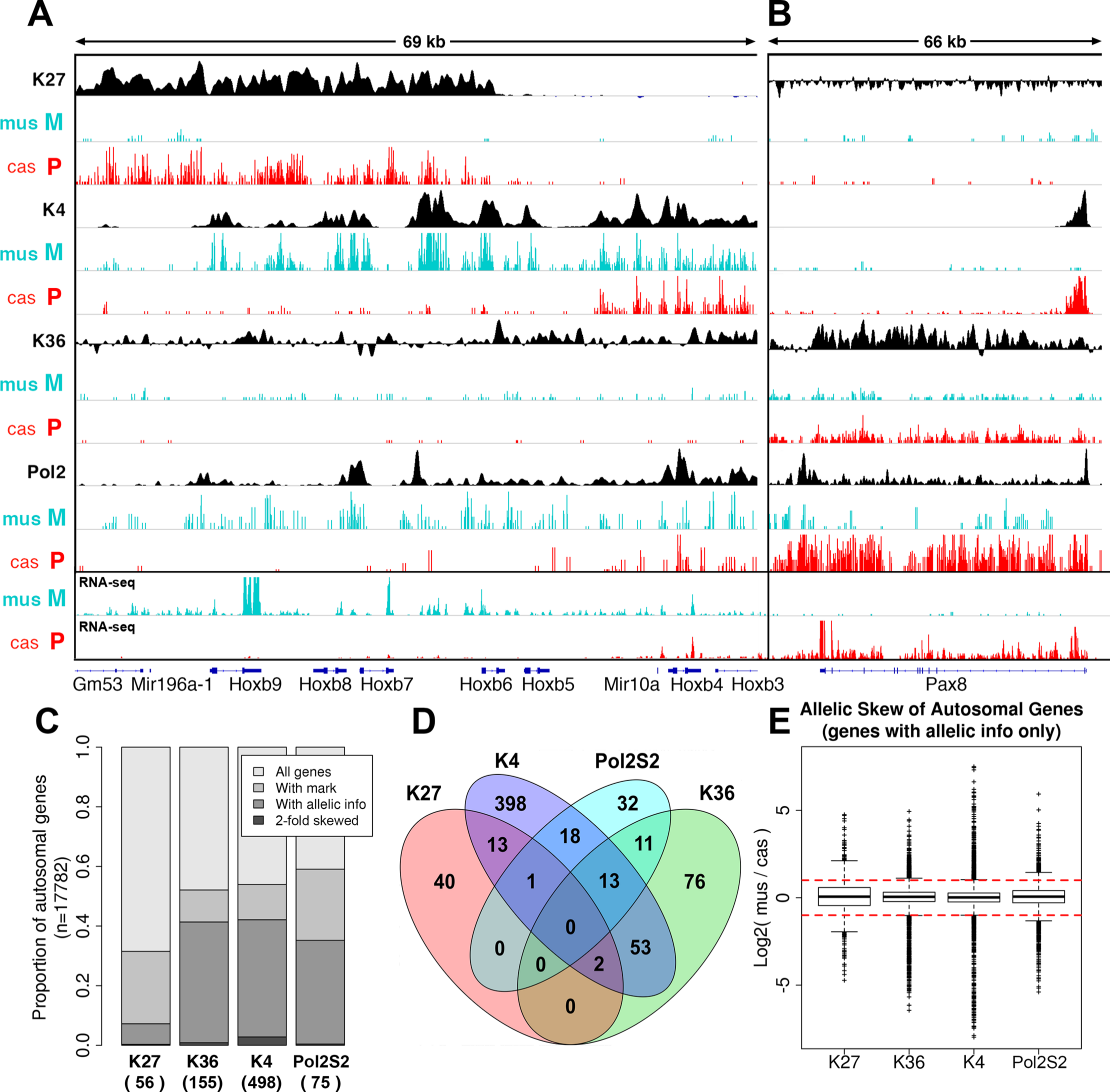
Allelic skew in chromatin state was detected in small but significant fraction of autosomal genes. **A, B,** Allelic density profiles of H3K4me3, H3K36me3, POL2-S5P, and H3K27me3 separated into paternal (red tracks, *cas*) and maternal alleles (cyan tracks, *mus*) marked as P and M, respectively. Composite densities based on all mapped reads are shown in black. **A,** *HoxB* cluster with maternally skewed expression. **B,** *Pax8* gene with paternally skewed expression. **C,** Numbers of autosomal genes with detected allelic skew in each individual mark (black, numbers indicated in parentheses), as a subset of all genes where this mark is present (“with mark”, gray), and of genes where the amount of allele-specific reads is sufficient to analyze allelic skew (“with allelic info”, dark gray). **D,** A Venn diagram showing the overlap between sets of genes skewed in K27me3, K4me3, Pol2S2, and K36me3 marks. **E,** Distributions of allelic skew values for individual marks, shown as boxpots of maternal:paternal (*mus/cas*) ratios of allelic read densities in log2 scale.

While the majority of chromatin mark densities on autosomal genes had an allelic skew well within a 2-fold range (Fig 1E), some exhibited a significant skew of two-fold or more in 0.3–2.8% of genes (Fig 1C), with a total of ~6.5% of genes with sufficient number of allelic reads showing a skew in at least one chromatin mark (S2 Table). This number corresponds to 3.7% of all autosomal genes. A fraction of these genes showed simultaneous skew in two or more marks (Fig 1D, Panels C and D in S1 Fig).

A notable example of allelic skewing is the *HoxB* cluster of homeobox genes–genes encoding transcription factors involved in differentiation, homeotic patterning, and development of various cancers. Within the *HoxB* cluster, we discovered 5 contiguous genes, *Hoxb5* to *Hoxb9*, with allelic skew in the same direction (Fig 1A). These genes showed consistent patterns of maternal skew in the densities of K4me3, K36me3, and POL2S5 (FDR = 5.1e-5), and paternal skew in the density of K27me3 (FDR = 2.3e-9). This pattern differs from other genes in the near vicinity, including other *HoxB* genes, which are separated from *Hoxb5* to *Hoxb9* by flanking miRNA genes, *mir196a-1* and *mir10a*. This is interesting, given that miRNAs have been linked with *in trans* control of *Hox* gene expression [36]. Another interesting feature of this region is the presence of high and strongly skewed K4me3, K27me3, and Pol2 occupancies in intergenic regions relatively distant from annotated promoters. For example, between *Hoxb6* and *Hoxb7* is a domain that is sharply marked by K4me3 on the maternal allele and by K27me3 on the paternal allele (Fig 1A), suggesting the presence of an unannotated transcript that is co-regulated with neighboring coding genes. Taken together, our data show for the first time that members of the *HoxB* cluster can be subject to allelic regulation, at least in this specific cell line.

As an example of more localized allelic skewing in the opposite direction, K4me3 was significantly skewed to the paternal allele at the transcription start site (TSS) of *Pax8* (Fig 1B, FDR = 4.7e-34). The TSS-proximal density of K4me3 at the paternal allele was ~7-fold higher than the density at the maternal allele. Correspondingly, POL2 was also skewed to the paternal allele along the gene body with approximately 7-fold ratio (FDR = 7.7e-53, Fig 1B). These skews were consistent with a 6.9-fold paternal skew of gene expression according to RNA-seq.

Gene sets with allelic skew partially yet significantly overlap with known imprinted genes (total 10 out of 25 imprinted genes [37] with sufficient number of allelic reads, hypergeometric *P*-value of 1.8e-06). This incomplete overlap is expected, given that many known imprinted genes demonstrate parent-of-origin-specific expression patterns only in a specific tissue or cell type.

Gene sets with allelic skew of chromatin state showed enrichment in several functional categories, as assessed by DAVID functional annotation tool [38]. These categories include chromatin-associated proteins such as histone clusters 1 and 2 (FDR of 8.2e-5 for K4me3) and proteins involved in chromatin modification such as EED, RBBP4, and CENPA. Genes with allelic skew in K4me3 occupancy are also enriched in mitochondrial proteins (FDR of 1.4e-4) and regulatory zinc finger proteins (FDR of 1.4e-4), whereas genes with allelic skew in POL2 are enriched in genes associated with alternative splicing (FDR of 6.2e-2). Consistent with previously reported analyses of monoallelic genes [19–22], we also observed genes involved in cell-cell recognition, signaling, and neurodevelopmental processes. An interesting example is provided by protocadherins. These neural cell adhesion proteins have been hypothesized to serve as cues in the development and maintenance of the large variety of neuronal identities and synaptic connections in the central nervous system [39]. Three major protocadherin gene clusters on chromosome 18 are organized somewhat similarly to immunoglobulin gene clusters, with the diversity of produced proteins achieved through combinatorial expression of variable exons and cellular specificity determined by allelic exclusion within individual neurons. Our data show allelic imbalance of histone marks within the alpha protocadherin cluster (*Pcdha3-4*, S2 and S3 Tables), the beta cluster at lower coverage levels (*Pcdhb-1*, *15*), and *Pcdh15* (S2 and S3 Tables).

### Relationships between levels of different marks on the same allele

**Fig 2:**
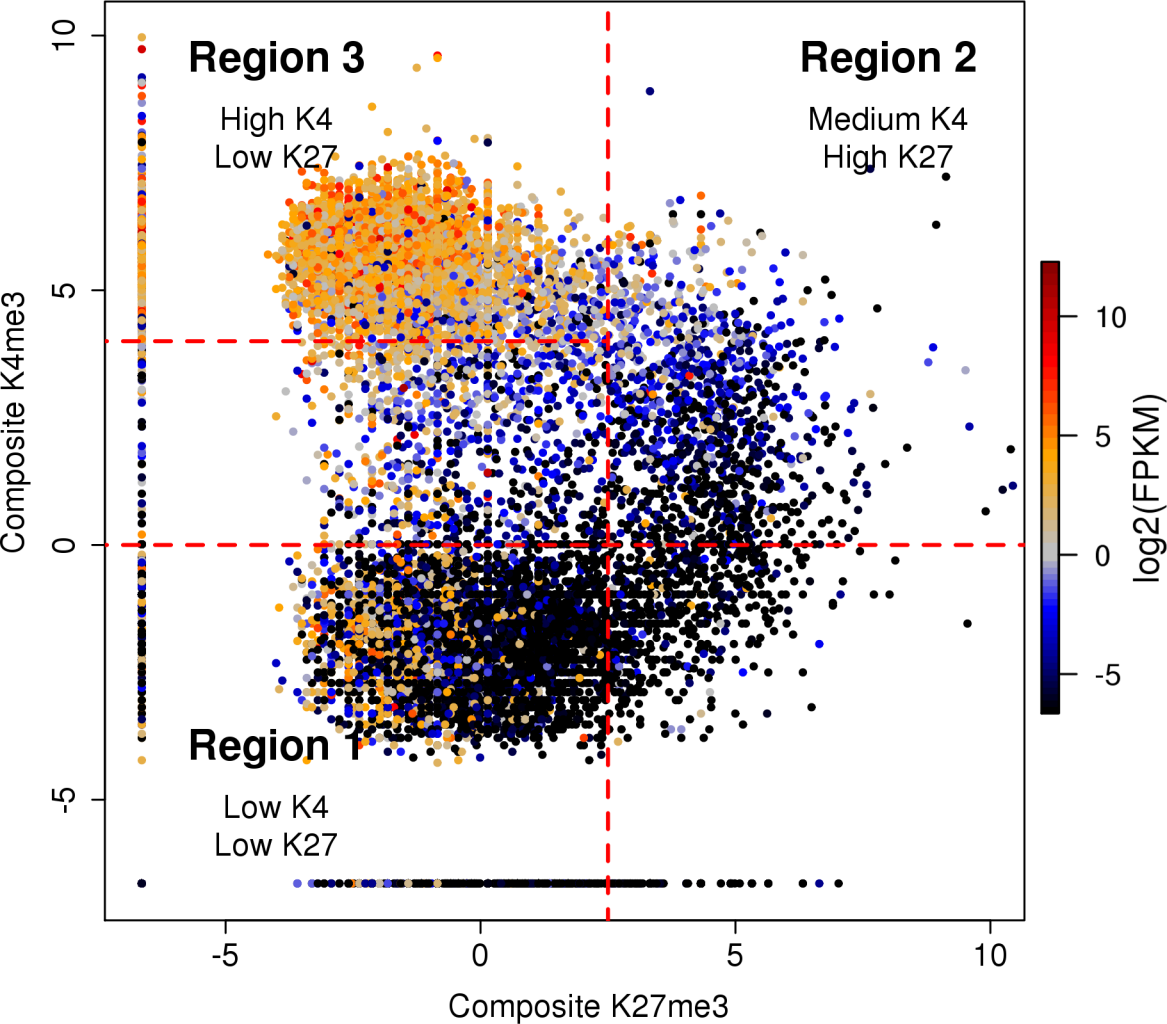
Quantitative relationships between active (K4me3) and repressive (K27me3) chromatin marks on the same promoter. Input-normalized densities of K4me3 and K27me3 in TSS-proximal regions of individual autosomal genes are shown in a two-dimensional scatter plot in log-log space, with color (black to red) representing levels of gene expression (silent to strongly active) measured by FPKM values based on RNA-Seq. Three major regions of the plot correspond to distinct levels and correlation patterns of K4me3 and K27me3. These regions largely correspond to silent (region 1), bivalent (region 2) and active genes (region 3).

Consistent with our previous results [11], the relationships between composite densities of K4me3, K36me3, K27me3, and POL2 at promoter regions involve three major types of chromatin state (Fig 2, S5 Fig). Permanently silent genes (Region 1 in Fig) show concerted depletion of both active and repressive marks. Transcriptionally active genes (Region 3 in Fig 2) have intermediate to high levels of active marks K4me3 and POL2 on promoters (S5 Fig), and K36me3 and POL2 on gene bodies (S5 Fig), combined with low to intermediate promoter levels of repressive mark K27me3. Importantly, this gene set shows a general anticorrelation between levels of active and repressive marks (Fig 2). A separate type includes transcriptionally inactive genes with the highest levels of K27me3, intermediate levels of K4me3, and general depletion of K36me3 and POL2 [11] (Region 2 in Fig 2, see also S5 Fig). This category largely corresponds to bivalent promoters, originally defined as promoters occupied by both K27me3 and K4me3 in embryonic stem cells [40]. Similar to bivalent genes in other differentiated cell types, bivalent genes in this MEF line were strongly enriched in key early developmental regulators. Analysis of functional set enrichment using DAVID [38] revealed strong enrichment in the categories of developmental proteins (Benjamini-Hochberg of 2.6e-36), homeobox genes (FDR of 7.5e-35), fork head transcription factors (FDR of 3.6e-5), embryonic skeletal system morphogenesis (FDR of 2.5e-6), and other gene sets related to development and cell differentiation. This enrichment was similar to the one observed in embryonic stem cells where bivalency was originally reported [40].

**Fig 3:**
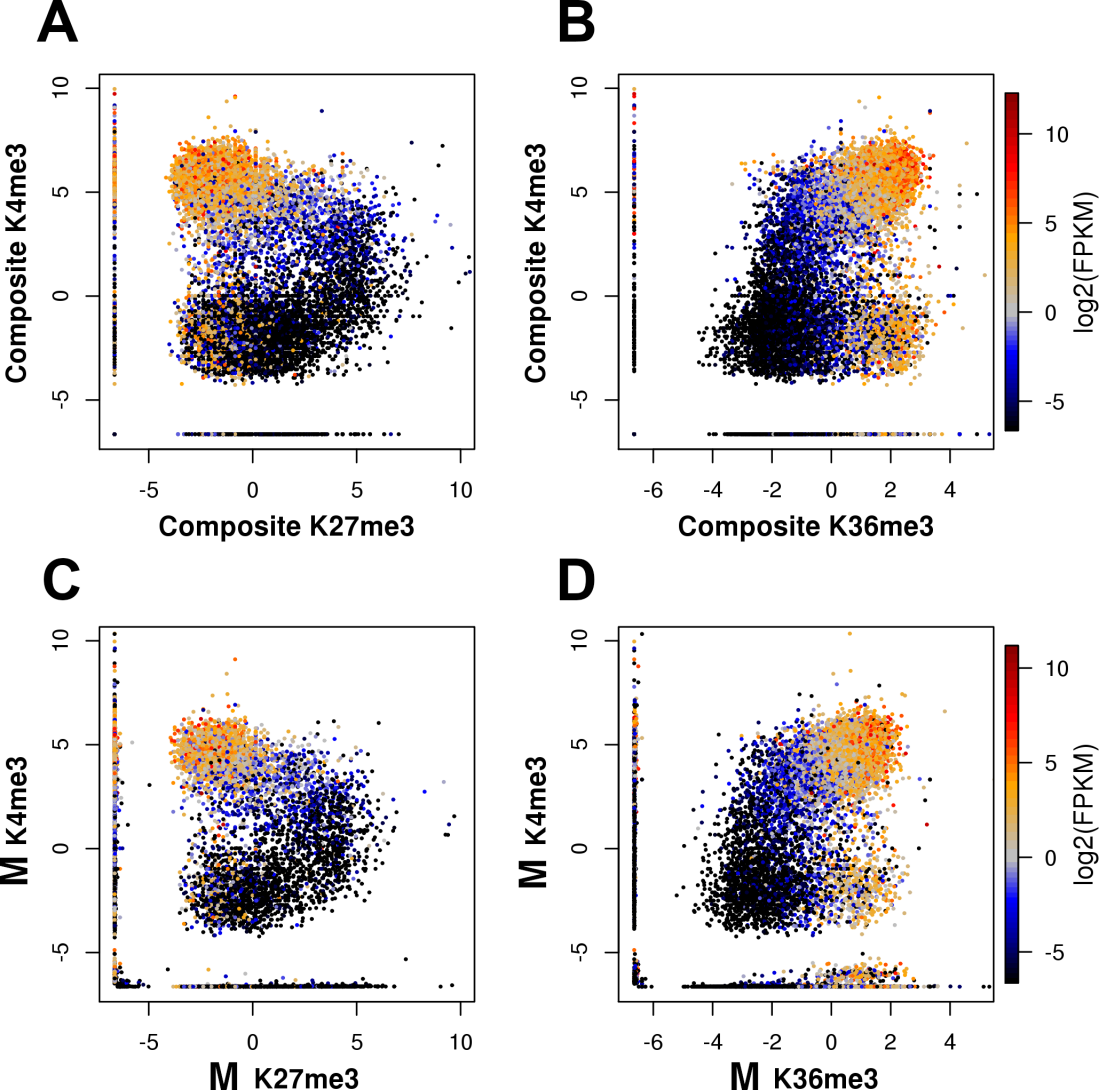
Relationships between chromatin marks on individual alleles are similar to the relationships on the composite level. **A, B**, Scatter plots of composite densities of K4me3 in TSS-proximal regions vs. (A) K27me3 in TSS-proximal regions and (B) K36me3 on gene bodies. Color represents composite levels of gene expression measured by FPKM values based on RNA-Seq. Active genes (upper part of the plots) show higher levels of active marks K4me3 and K36me3, general anticorrelation between K4me3 and K27me3, and positive correlation between K4me3 and K36me3. Permanently silenced genes (lower part of the plots) show depletion of K4me3 and K36me3, and the corresponding depletion of K27me3. Bivalent region is the area of highest K27me3 densities and intermediate K4me3 densities that connects active and permanently silenced branches in (A). **C, D,** Scatter plots of inferred mark densities on individual allele (maternal “M” allele as an example) for K4me3 in TSS-proximal regions vs. (C) K27me3 in TSS-proximal regions and (D) K36me3 on gene bodies. Color represents allelic levels of expression measured by FPKM values based on RNA-Seq.

The relationships between the inferred *allele-specific* levels of chromatin marks are remarkably similar to the relationships between their composite levels (Fig 3, S6 and S9 Figs). Individual alleles can be roughly categorized into the same three major types: active, permanently silent, and bivalent. In the case of bivalent alleles, high levels of K27me3 and moderate levels of K4me3 co-occur on the same allele, suggesting that bivalency, which was initially described on the composite level, is not a combinatorial effect of mark densities on different alleles (Fig 3, S6 and S9 Figs). This observation is consistent with the reported co-occurrence of repressive and active marks on the same nucleosome and even histone tail [41,42].

Among genes with allelic skew, distributions in the skew magnitude often depend on the gross type of chromatin state. S7 Fig shows the distributions of composite expression values (Panel A in S7 Fig) and distributions of allelic skews of K4me3, K27me3, and K36me3 (Panels B-D in S7 Fig) for all autosomal genes and for separate gene types (permanently silent, bivalent, and active) corresponding to regions 1–3 in Fig 2. These types show different levels of expression, from low to intermediate to high (Panel A in S7 Fig). In each type, the observed allelic skews of chromatin marks are symmetrically distributed around zero, suggesting the absence of systematic skew towards either maternal or paternal allele (Panels B-D in S7 Fig). However, the ranges of allelic skews can be different among these gene types: as expected, a low or absent histone mark density is associated with the low likelihood and extent of allelic imbalance. For example, K4me3 and K27me3 are low or depleted among silent genes (Region 1 in Fig 2), which results in lower ranges of skew magnitude in this region (Panels B and C in S7 Fig), whereas higher composite enrichment of a mark (e.g. K4me3 in active region 3 in Fig 2) allows for stronger allelic skews (Panel C in S7 Fig). Notably, bivalent promoters (Region 2) are not enriched for allelic skew. The vast majority of these promoters have a skew near zero, as shown by an extremely narrow width of the box in the boxplots of allelic skew in region 2 (Panels B and C in S7 Fig), which corresponds to the range between the first and third quartile of the statistical sample. These quartiles correspond to near-zero skew values, suggesting that bivalent promoters largely do not have allelic skew in either K27me3 (Fig 2B) or K4me3 (Fig 2C), with the exception of a few outliers (shown as crosses). Thus, at the vast majority of bivalent promoters, high K27me3 and moderate K4me3 levels are present on both individual alleles, as opposed to the combination of one K27me3-only and one K4me3-only allele.

### Combinations of allelic chromatin states observed in skewed genes

**Fig 4:**
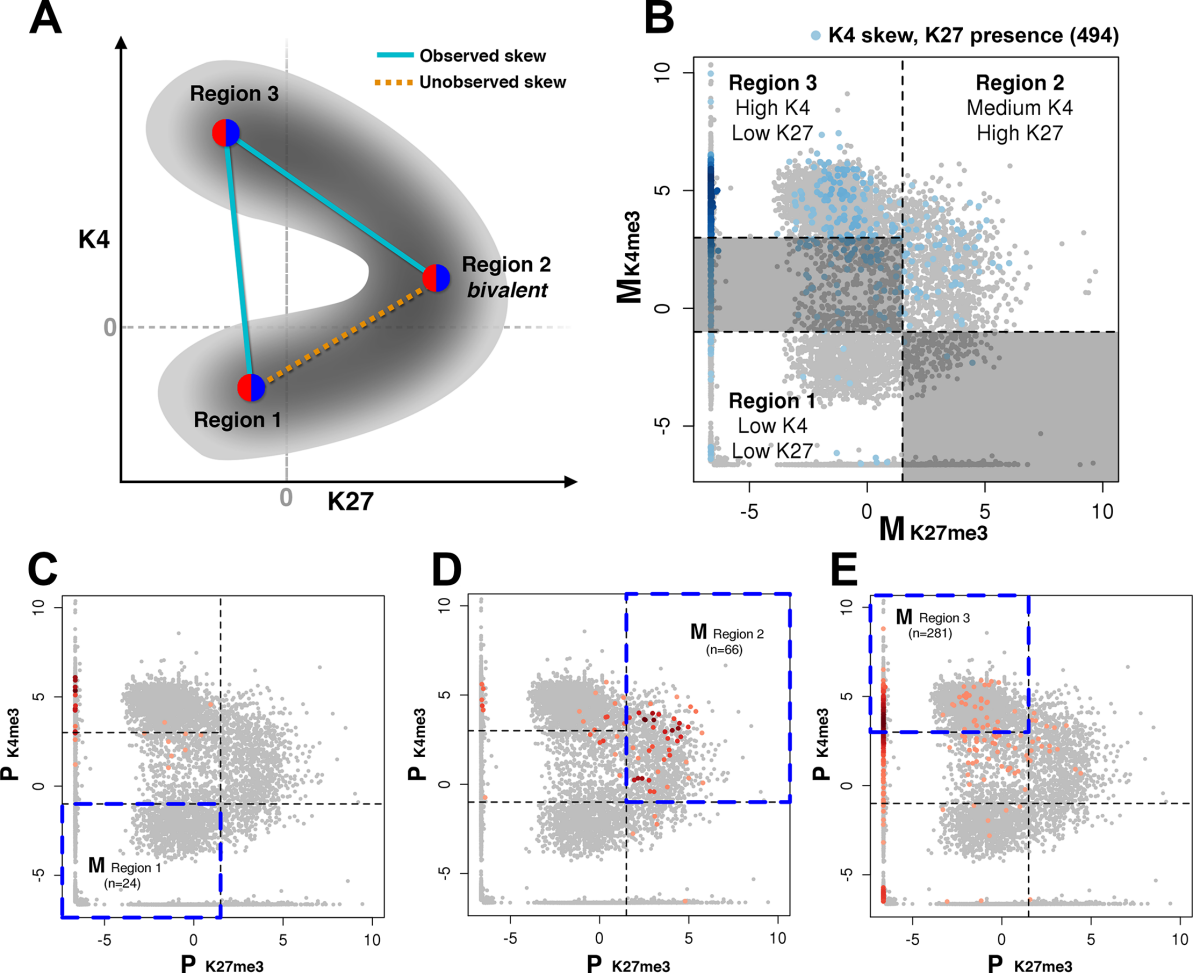
Pairwise combinations of allelic chromatin states that result in observed allelic skew of a chromatin mark. Allelic promoters are shown as points in the space of K4me3 and K27me3 densities. **A,** Schematic of major types of allelic chromatin states and their possible combinations on two alleles. Regions 1, 2, and 3 correspond to silent, bivalent, and active alleles, respectively. Combinations observed in larger numbers of genes (“observed”) are shown as cyan lines. Combination observed in fewer genes (“unobserved”) is shown as orange line. **B,** Scatter plot of K4me3 vs. K27me3 promoter densities on maternal (M) *mus* allele of all autosomal genes shown as gray points; genes with allelic skew in K4me3 are highlighted in blue. Regions 1, 2, and 3 are marked by dashed lines. **C-E,** Scatter plots of K4me3 vs. K27me3 promoter densities on paternal (P) *cas* allele, shown as gray points. Red points in these three plots highlight three subgroups of genes shown in **B**: genes whose maternal allele belongs to silent, bivalent, or active type (regions 1, 2, and 3 in **B**, marked for the reference by a blue rectangle in each corresponding plot **C-E**). Hue indicates the local density of paternal alleles with similar K4me3/K27me3 densities. **C,** Among genes with K4me3 skew whose maternal allele is in a silent state (region 1 in **B,** *n* = 24), paternal allele (P) generally has medium to high K4me3 density and low K27me3 density. **D,** Among genes with K4me3 skew whose maternal allele is in a bivalent state (region 2 in **B,** *n* = 66), paternal allele (P) generally also resides in a bivalent state. **E,** Among genes with K4me3 skew whose maternal allele is in an active state (region 3 in **B,** *n* = 281), paternal allele (P) most often resides in an active or bivalent state; there are much fewer cases of a fully silent paternal allele.

We next examined the relationships between pairs of chromatin marks on genes with allelic skew. Fig 4 shows the observed combinations of K4me3 and K27me3 densities on different alleles of genes with K4me3 skew. Schematic in Fig 4A shows three major types of allelic chromatin states in the space of K4me3 and K27me3 promoter densities, with pairwise combinations of allelic states of different types represented by lines. Two alleles can reside either in the same or in different regions of this plot. Focusing on the autosomal genes with K4me3 skew, Fig 4B shows the observed locations of one (maternal) allele of these genes (blue points). These alleles occupy mostly active (Region 3, *n* = 281) and bivalent states (Region 2, *n* = 66), with the rest genes occupying region 1 and the area between regions 1 and 3. Since we focused on the relationship between K4me3 vs K37me3 levels, we did not include *n* = 4 promoters without any H3K27me3 reads, which reduced the number of considered promoters to 494 from the total of 498 promoters with allelic skew in K4me3 (Fig 1C).

Fig 4C–4E show the locations of the other (paternal) allele that correspond to the maternal allele residing in the silent region 1 (Fig 4C), bivalent region 2 (Fig 4D), or active region 3 (Fig 4E). In most genes, with the exception of silent alleles (Fig 4C, *n* = 24), both alleles belong to the same major type of chromatin state and reside in the same corresponding region of the plot (Fig 4D and 4E). In genes that have one silent allele with depleted K4me3, the allelic skew results from the other allele having a higher level of K4me3, which corresponds to a chromatin state outside of region 1 (Fig 4C). In general, however, the cases of genes with two alleles in different major categories are much less frequent. A similar trend was observed for the combinations of allelic states in K4me3/K36me3 space (S5 Fig), suggesting that despite a relatively widespread occurrence of allelic imbalance, two alleles of a gene are rarely in different types of chromatin states. For example, on the majority of bivalent promoters both alleles are in the bivalent state, reinforcing the notion that K4me3 and K27me3 have a largely balanced presence on both alleles (S7 Fig) and thus co-occur on the same allele. S9 Fig highlights allele-specific combinations of marks (K4me3 vs K27me3 and K4me3 vs K36me3) at bivalent genes (green points). For the majority of promoters that are bivalent at the composite ChIP-seq level (green points in Panels A and B in S9 Fig), each individual allele is also bivalent, with the corresponding points in allelic plots (Panels C and D in S9 Fig for *mus* and Panels E and F in S9 Fig for *cas*) located in the same region of high levels of K27me3, moderate levels of K4me3, and mostly depleted K36me3.

Among the genes with alleles in different types of chromatin states, particular combinations of these states are more frequent than others (Fig 4B–4E). Interestingly, there is an underrepresentation of promoters with one bivalent and one silent allele (“unobserved skew” in Fig 4A, S11 Fig). The apparent barrier between these two types of states is consistent with the previously observed low likelihood of promoter transitions between bivalent and silent states during ESC differentiation [11], suggesting that there may be a molecular mechanism that makes unlikely a transition between bivalent and silent state (Fig 4A, dashed orange line). Bivalent promoters are occupied by a variety of protein complexes, including PRC1, PRC2, and SET1/MLL, which are involved in intricate interactions with chromatin and each other in order to precisely regulate key developmental genes that may be activated during differentiation [43,44]. On the other hand, promoters of permanently silent genes are characterized by depletion of POL2, K4me3, K36me3, and K27me3 (Fig 3A and 3B) consistent with transcriptional repression via more permanent alternative mechanisms (H3K9me3 methylation, DNA methylation, long-term chromatin compaction, etc.) [45]. These differences may serve, at least in part, as a mechanistic explanation of the barrier between the two states.

### Relationships between allelic skews of different marks at the same gene

**Fig 5:**
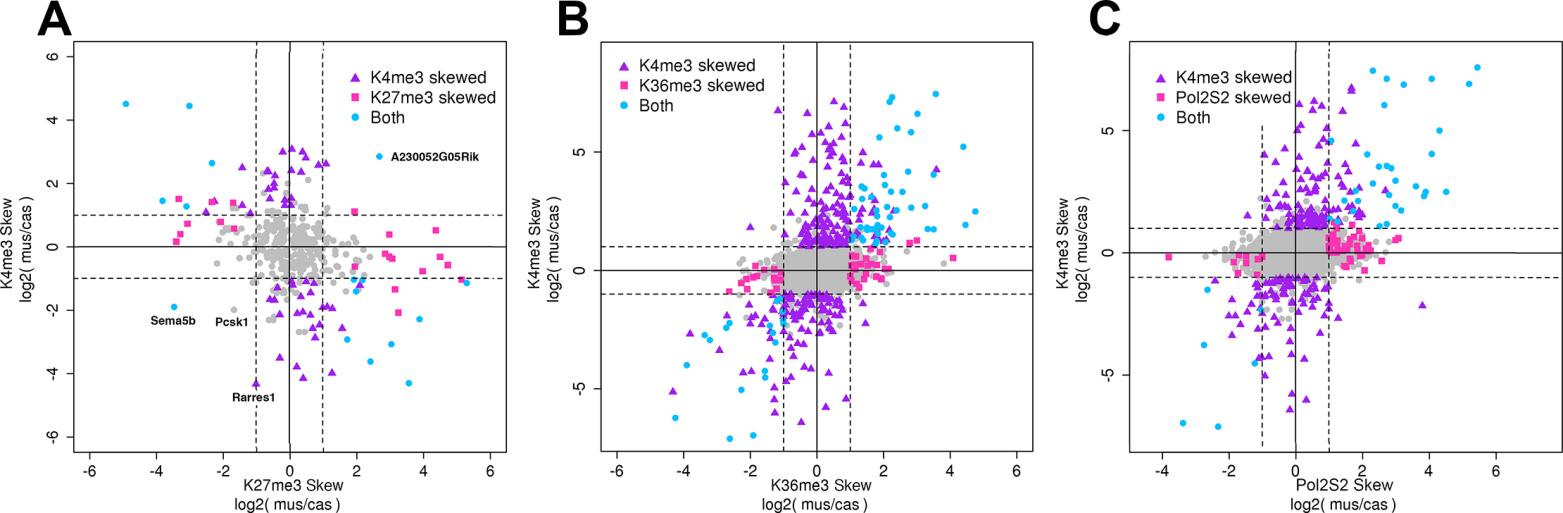
Relationships between the magnitudes of allelic skew of different marks on the same gene. For all genes with sufficient number of allelically assigned reads, allelic skews (log2 of *mus*:*cas* ratio) are plotted for one mark vs. another: **A,** K27me3 vs. K4me3; **B,** K36me3 vs. K4me3; **C,** POL2S2 vs. K4me3. Horizontal and vertical dashed lines mark the 2-fold allelic difference. The majority of genes do not show significant skew in any mark (gray points around origin). Most of the genes with skewed chromatin state (colored points) have a skew in only one mark and no significant skew in the other (purple triangles and magenta squares). However, when both marks are skewed (cyan circles), these skews are anticorrelated for active vs. repressive mark (A) and correlated for two active marks (B, C).

We next compared the degrees of allelic imbalance of individual chromatin marks at the same gene. Fig 5A shows the comparison between maternal:paternal (*mus*:*cas*) ratios of K4me3 and K27me3 densities on promoters where at least one of these marks is skewed. This comparison leads to two main observations. First, most of these promoters are skewed in only one mark, suggesting the absence of a general association between the allelic imbalances of different histone marks. Second, in an apparent contradiction, when considering the genes that have allelic skews in both marks, these skews are anti-correlated: if K4me3 density is skewed towards one allele, K27me3 density is skewed towards the other (Fig 5A). We observed only a handful of exceptions where K27me3 and K4me3 are skewed towards the same autosomal allele (*Rarres1*, *A230052G05Rik*, *Pcsk1*, and *Sema5b*, marked in Fig 5A). These four genes correspond to the rare combination of one allele in the “silent” chromatin state, with the depletion of both K4me3 and K27me3 (Region 1 in Fig 4A), and the other allele in or near the region corresponding to “bivalent” state, with the presence of both K27me3 and K4me3 (Region 2 in Fig 4A). As a result, all these genes are transcriptionally inactive (composite FPKM < 0.1).

The relationships between allelic skews of active marks K4me3 and K36me3 (Fig 5B), K4me3 and POL2 (Fig 5C), and K36me3 and POL2 (S10 Fig) show a similar pattern: many genes have a skew in only one mark, but if both active marks are skewed, the directions of these skews are positively correlated. S4 Table shows pairwise correlations between allelic skews of all marks, for the genes where both marks are skewed.

This apparent contradiction can be explained by taking into account the relationships between histone marks on the same allele (Fig 3C and 3D, S6 Fig) and possible combinations of chromatin states of the two alleles. These combinations are schematically shown in Fig 4A as pairs of points in the continuous space of K4me3 and K27me3 densities, and in S11 Fig as a table of pairwise combinations of major types of states (permanently silenced, bivalent, and active). Considering relationships between K4me3 and K27me3 densities as an example, the combination of two silent alleles occupying different locations within region 1 (Fig 4A, S11 Fig) corresponds to a strong depletion of K4me3 on both alleles and thus does not allow for a skew in K4me3. This combination can result only in the skew of K27me3. The combination of one silent (region 1) and one active allele (region 3) corresponds to low or modest levels of K27me3 on both alleles and thus does not allow for a strong skew in K27me3 (Fig 4A, S11 Fig). This combination can result only in the skew of K4me3.

The simultaneous skew in both marks can be produced by either the combination of two active alleles at different locations within region 3, or the combination of one bivalent and one active allele (regions 2 and 3, tilted cyan line in Fig 4A). Since region 3 (active alleles) is characterized by a pronounced anticorrelation between K4me3 and K27me3, these two imbalanced alleles will have opposite mark levels: high K4me3 and low K27me3 on one allele vs low K4me3 and high K27me3 on the other (Fig 4A). These opposite levels of K4me3 and K27me3 on the two alleles lead to the opposite allelic skew of K4me3 compared to K27me3.

Thus for imbalanced alleles with a simultaneous skew of two chromatin marks, the apparent coordination between skews of these marks (Fig 5) is a result of quantitative relationships between different chromatin marks on the same allele (Fig 3C and 3D, S6 Fig).

### Allelic chromatin states quantitatively predict allelic expression

To assess the relationship between allelic skews of chromatin state and gene expression, we performed allele-specific RNA-seq in the same hybrid cell line. Consistent with higher sequence conservation in coding transcripts, the fraction of reads that could be assigned to a specific allele in RNA-seq experiments (approximately 17%, Panel A in S1 Fig and S12 Fig) was lower than in ChIP-seq experiments. When examining allele-specific levels of RNA expression, we observed a distinct and reproducible allelic skew in a subset of autosomal genes. For example, *Hoxb5-9* and *Pax8* genes (Fig 1A and 1B) show allelic skew in expression. Generally, approximately 50% of genes contained enough allele-specific reads to reliably calculate an allelic expression skew. Similar to our ChIP-seq based results, we detected 5.6% of genes with a significant skew in expression of two-fold or more (S5 Table).

**Fig 6:**
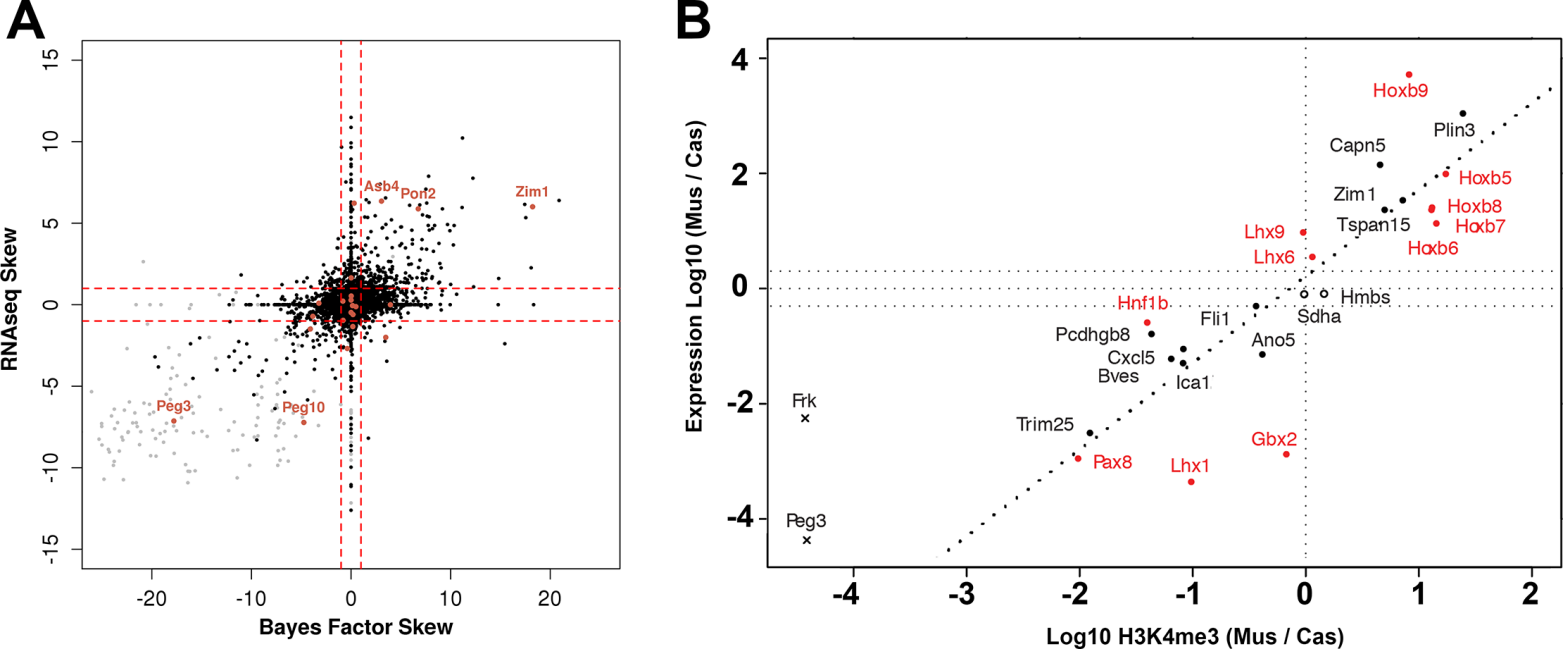
Allelic skew of epigenetic state predicts allelic skew in gene expression. **A,** Comparison of the skew in allelic expression (log2 of *mus*:*cas* ratio) predicted from allelic densities of histone marks and POL2 to the observed skew based on allele-specific RNA-Seq. Black points, autosomal genes; gray points, X-linked genes. Known imprinted genes with predicted/observed skew are highlighted in orange. Horizontal and vertical dashed lines mark the 2-fold allelic difference. **B,** Allelic skew in gene expression, as determined by qRT-PCR, is strongly associated with skew in H3K4me3. Both axes are in decimal logarithm scale. Linear fit is shown as dashed line (*R*^2^ = 0.70). Filled circles: selected genes with allelic skew of various marks. Empty circles: control housekeeping genes with no skew. Red: homeobox proteins. Crosses: genes with zero K4me3 coverage on one allele, which makes the estimate of skew less precise. Horizontal dashed lines mark the 2-fold allelic difference.

Combinations of composite levels of chromatin marks are known to be quantitatively predictive of composite gene expression levels [6–10,34]. Here, we performed a similar analysis at allele-specific resolution and addressed the questions of (a) whether the correlation between chromatin marks and gene expression holds for individual alleles; and (b) whether allelic skew in gene expression can be quantitatively predicted from allelic levels of chromatin marks. First, we found that allelic levels of individual chromatin marks are correlated with allelic expression: levels of active marks K4me3, K36me3, and Pol2 on a particular allele correlate with expression of that allele, whereas levels of repressive mark K27me3 are anti-correlated with expression. Second, to test whether allele-specific skew in chromatin state is correlated with allelic-expression skew (RNA-seq), we constructed a Bayesian model that quantifies overall chromatin skew for all surveyed marks (see SI Methods). After training, the resulting model achieved a correlation with expression skew of *R* = 0.57 (Fig 6) for all genes with allelic coverage and *R* = 0.64 when limited to expressed genes (composite FPKM > 1). Thus imbalance of chromatin mark densities between alleles corresponds to imbalance in the expression of these alleles. S11 Fig schematically shows pairwise combinations of major types of allelic chromatin states (active vs silent, active vs bivalent, active vs active) that can be associated with a skew in gene expression.

Different chromatin marks contribute unequally to expression skew. To test their separate and combined predictive power, we performed linear modeling in a simple 2-fold cross-validation framework. Expression levels predicted from a combination of levels of chromatin marks correlated moderately with observed expression levels from a given allele (mean *R* = 0.51±0.02). K4me3 and K36me3 skews individually explain the largest fractions of expression skew variance (S1 Table), and the combined model provided a better prediction of allelic expression skew than individual marks based on a range of various skew cutoffs (S13 Fig). As an example, the expression of *Hoxb5*, *6*, and *7* genes shown in Fig 1A is strongly predicted as maternally biased, with the predicted skew values of 1.4, 4.7, and 2.2, respectively. This prediction is confirmed by observed RNA-seq skews of 4.4, 6.0, and 3.7, respectively.

A smaller number of genes had discordant skews in chromatin state and expression. The set of 37 genes (0.2% of total or 1.0% of expressed autosomal genes with allele-specific ChIP-seq reads) showed anticorrelation between predicted and observed allelic skews in expression (Fig 6A). Another set of genes had a significantly skewed chromatin state but showed apparent absence of significant expression skew (Fig 6A). Finally, a group of 256 genes (1.4% of all autosomal genes, 6.9% of expressed autosomal genes with allele-specific ChIP-seq reads) had allelic balanced chromatin state but showed allelic skew in RNA-seq (Fig 6A). Interestingly, these genes were strongly enriched in histones (DAVID FDR = 6.3e-11), ribosomal proteins (DAVID FDR = 1.2e-5), and ribonucleoproteins in general (DAVID FRD = 4.4e-10). The inspection of of ChIP-Seq enrichment of chromatin marks at many of these genes showed no significant presence of histone marks at either TSS or gene body. This is consistent with our previous observations for histone genes in particular, suggesting an intriguing possibility of alternative regulatory mechanisms at these strongly expressed essential genes that maintain high levels of transcription throughout most stages of cell cycle.

More generally, some of the observed inconsistencies between allelic skews of chromatin state and gene expression may suggest regulation by factors outside the four surveyed marks. A part of this variation may be due to post-transcriptional effects, since genotypic skewing is often associated with higher variant density in transcript termini, potentially affecting transcript stability [16]. Other inconsistencies may be due to lower numbers of reads with allelic assignments and could be resolved with additional depth of allelic sequencing.

To assess the role of DNA polymorphisms between the two alleles and compare this role to sequence-independent effects in allelic expression, we analyzed public RNA-seq data in MEFs from inbred mouse strains of Mus musculus and Mus castaneus [16]. We compared differences in gene expression between inbred strains to the allelic skews observed in our hybrid cell line. Although genetic background of these strains may not be completely identical to that of parental strains used to produce our hybrid MEFs, this comparison may provide a rough lower-bound estimate of the role of sequence polymorphisms in the observed allelic skews. The levels of gene expression in the inbred *mus* and *cas* strains are largely very similar, but a fraction of genes shows substantial expression skew (greater than 2-fold difference, Benjamini-Hochberg FDR < 0.01; *n* = 2611, Panel A in S14 Fig). Focusing on the genes with adequate expression (FPKM > 1.0) in at least one inbred strain and at least one allele in the hybrid MEFs, we compared these differences in strain-specific gene expression to the allele-specific skew in the hybrid cells (Panel B in S14 Fig). We observed three major groups of skewed genes: (a) genes with allelic skew in hybrid cells that is consistent with the skew between inbred lines and thus is likely driven by genetic differences (*n* = 49); (b) genes that have no significant differences between inbred lines but show allelic skew in hybrid cells, which is possibly maintained through sequence-independent mechanisms (*n* = 181); and (c) genes that show expression difference between inbred lines but do not show allelic skew in the hybrid (*n* = 415), which may be attributed to potential variability between cell lines, or to the less likely coordination between expression from individual alleles in the hybrid.

As expected, expression of the vast majority of X-linked genes is predicted and confirmed as paternally skewed (Fig 6A, gray points), consistent with the inactivation of maternal X chromosome in this female cell line.

Additional validation by allele-specific RT-PCR confirmed the observed expression skew for a number of tested genes (Fig 6B). Consistent with RNA-seq results, K4me3 was the strongest quantitative predictor of skewing at the expression level (*R*^*2*^ = 0.70) across a wide range of skew, from genes with no K4me3 skew in control genes (empty circles), to moderate skew, to extreme skewing (upper and lower corners of the plot).

Together, our findings lead to several conclusions. First, we were able to identify a number of autosomal genes with significant allelic skew in chromatin state. Second, in the majority of these genes, the skew is produced by quantitative differences of histone mark densities between two alleles in the same major type of chromatin state (active, bivalent, or silent). Genes with two alleles in different types of chromatin state are less frequent. Third, when two histone marks are skewed, these skews are coordinated as a result of the quantitative relationships between these marks on each individual allele. Finally, combination of allele-specific densities of chromatin marks is a quantitative predictor of allelic skew in gene expression.

Performing allele-specific ChIP-seq and RNA-seq experiments on large numbers of cultured cells is a conservative approach to the analysis of long-term allelic skews that are consistent among large cell populations. Here we observed a quantitative correlation between allelic imbalance in the chromatin state based on ChIP-seq experiments and allelic imbalance in gene expression based on RNA-seq. This consistency confirms the value of our approach for the analysis of both chromatin and expression allelic imbalances that are prevalent among the statistical ensemble of millions of cells. This approach, however, may be insensitive to transient allelic imbalances that occur in small subsets of cells or at shorter time scales. For example, our estimate of the extent of allelic imbalance in autosomal gene expression is more conservative than the estimate that RMAE occurs in 12–24% of individual fibroblasts based on single-cell RNA-seq [27,29–31].

This experimental approach has its obvious limitations. The analysis of individual alleles is confined to genomic regions that are sufficiently covered by allele-specific SNPs and indels. In spite of high average density of allele-specific SNPs in *mus*/*cas* hybrid cells, there are many loci where allele-specific information is unavailable. Previous allele-specific RNA-seq studies on large numbers of cultured cells show considerable variability of skews at specific loci across cell lines and strains [16]. Our study is focused on one cell line, and a similar analysis of chromatin states in the cell line from reciprocal mouse hybrid should help to further clarify the contributions of sequence-dependent and sequence-independent mechanisms to the observed allelic imbalances. A broader analysis of cell line- and strain-specific variability of allelic chromatin states will also be an interesting direction for future studies. However, even in the analysis of a single MEF line, comparing allelic skews in chromatin state and expression across all autosomal genes both provided a validation of these two different types of allelic data and gained new insights into the mechanisms of allelic imbalance. Among other findings, our results confirm that the phenomenon of bivalency is not the artifact of superposition of two distinct allelic states but occurs on each individual allele of a given promoter.

Our results leave multiple outstanding questions about genomic and epigenomic mechanisms and functions of allelic imbalances of chromatin states: the relative contributions of genetic and non-genetic (sequence-independent) mechanisms to these imbalances; the presence and ways to detect potential quantitative trait loci (QTLs) responsible for the allelic skews in chromatin marks; variability in allele-specific chromatin states between cell lines, strains, and stages of development and differentiation; variability of allelic imbalance between individual cells; potential short-term transitions or fluctuations of chromatin state of an individual allele in an individual cell through time. Addressing these and other questions in the future would be crucial for better understanding of regulatory mechanisms, biological functions, and clinical relevance of allelic imbalances in chromatin state and their relation to RMAE and other allele-specific phenomena.

## Materials and methods

### Cell line

To generate the clonal hybrid mouse embryonic fibroblast (MEF) cell line [34], female mice of *Mus musculus* (129S1) and male mice of *Mus castaneus* (CAST/EiJ) origins were crossed. Female F1 embryos were collected at day 13.5 and used to prepare MEFs. These cells were later immortalized by SV-40 T-antigen subcloned by limiting dilution.

### Calculation of allele-specific ChIP-seq coverage

Input-normalized allele-specific ChIP-seq read density was calculated based on previously published H3K4me3, H3K36me3, POL-II-S2P, POL-II-S5P ChIP-seq datasets (GEO GSE33823) by Yildirim et al [34] and H3K27me3 ChIP-seq dataset (GEO GSE36905) by Pinter et al.[33], accompanied by the corresponding input datasets. BWA aligner [46] with default parameters was used to map paired-end reads (17–28 million per sample) to two variant strain genomes (CAST/EiJ and 129S1/SvImJ) of the hybrid cell line, which were reconstructed from mm9 reference using catalogued SNPs and indels [35], with ~22 million allele-specific SNPs and ~1 million indels, approximately one modification per 120 bp. Uniquely aligned reads that had a higher mapping score to one of the two strain genomes were classified as allele-specific and assigned to the higher-scoring allele variant; otherwise they were classified as neutral.

Fragment density was calculated separately for allelic tracks (*mus*, *cas*) and composite track (allelic and neutral combined) based on fragments defined by paired reads (~400 bp on average), discarding duplicate fragments. Promoter regions were defined as segments including +/- 1 kb from annotated TSS. For each promoter region and gene body, numbers of mapped fragments in composite and two allelic tracks were counted. Allelic skew was estimated from allelic (*cas* and *mus*) read numbers mapped to each region. We then estimated statistical significance using a binomial *P*-value based on the assumption that allele-specific reads are randomly mapped to each strain with the probability of 0.5. These *P*-values were further corrected for multiple testing by calculating Benjamini-Hochberg false discovery rate (FDR). To identify regions of significant allelic skew, we used regions with ≥ 15 total allele-specific reads, higher than 2-fold difference between read numbers mapped to the two alleles, and FDR < 0.05. *Inferred* allelic coverage was estimated as a fraction of composite coverage corresponding to the observed *mus*:*cas* ratio of allelic reads.

### RNA-seq

Total RNA was isolated from hybrid female clonal cell line of MEFs (EY.T4) prepared from the cross of female mice of *Mus musculus* (129S1) and male mice of *Mus castaneus* (CAST/EiJ) origins [34]. After ribosomal RNA depletion with RiboMinus Eukaryote System v2 (Thermo Fisher Scientific), libraries were prepared by the dUTP-based method using NEBNext Ultra Directional RNA Library Prep Kit for Illumina (E7420) (NEB). Two biological replicates were sequenced on Illumina HiSeq2000 instrument, resulting in over 30 million reads per sample. Homozygous MEF expression values were computed from data retrieved from GEO series GSE58524.

### Estimation of levels and allelic skews of gene expression

Transcriptome mapping of RNA-seq reads was performed with STAR version 2.3.0 [47] using the 2-pass method described in Engström et al [48]. Read counts for individual genes were produced with HTSeq v.0.6.0 [49] using the unstranded, intersection-nonempty option with Ensembl release 67 transcript definitions. Allelic skew was estimated from allelic (*cas* and *mus*) read numbers mapped to the transcript. To identify transcripts with significant allelic skew in expression, we used regions with ≥ 15 total allele-specific reads, higher than 2-fold difference between allelic expression values, and Benjamini-Hochberg FDR < 0.05. *Inferred* allelic expression was estimated as a fraction of composite expression level corresponding to the observed *mus*:*cas* ratio of allelic reads.

### Bayes factor prediction of gene expression

We utilized Bayes Factor scores on training sets, a method analogous to one previously described [50], in order to predict allele specific gene expression (S7 Fig). Briefly, genes were divided into equal sized training and validation sets. For each ChIP-seq experiment, the distributions of enrichment over gene regions for the expressed and non-expressed genes were determined. For each allele for each gene, a Bayes Factor was calculated, representing the log likelihood that the observed ChIP-seq enrichment value was drawn from either the expressed or non-expressed reference distributions. Log Bayes Factors were summed across all ChIP-seq sets for a final value.

### Allele-specific qRT-PCR

RNA was isolated using TRIZOL Reagent (Invitrogen). cDNA was then generated with SuperScript III reverse transcriptase (Invitrogen) and oligo(dT)15 primer (Promega), and was subsequently used for qPCR. 20-μl reactions were run in technical duplicate on 96-well plates using 250 nM each of universal and either Cas- or Mus-specific primer, and SYBR Green supermix (Bio-Rad). The PCR program consisted of 45 cycles of: 95°C, 15 sec; 60°C, 30 sec; 72°C, 30 sec. Primers targeting differential SNPs between alleles were designed according to the method of TaqMAMA [51]. Specifically, we aimed to target exonic regions containing ≥2-nt difference between alleles within a 4-nt span. For genes in which this does not occur or does not easily lend itself to primer design, single mismatches were intentionally placed at the nucleotide directly 5’ to individual SNPs to achieve higher sensitivity. The identity of the mismatched base was chosen to optimize allelic discrimination, as previously described [52]. Expression levels were compared against that of the opposite allele using the formula: Fold Difference = 2^Ct(*mus*-*cas*).

## Supporting information

S1 Fig Overview of quantifying allelic imbalance in gene expression and chromatin marks. **A.** ChIP-seq and RNA-seq reads that overlap *mus*/*cas* SNPs (ChIP-seq approx. 37%, RNA-seq approx. 17%, of total reads) can be assigned uniquely to one parental allele: *mus*, maternal; *cas*, paternal.
B. A methodology schematic showing the calculation of allelic densities and expression for ChIP seq and RNA seq reads, respectively.
C. A fraction of genes showed simultaneous allelic skew in two or three marks. The barplot shows the numbers of genes with simultaneous skew in 2 or 3 marks as fractions of genes with skew in each individual mark (K27me3, K36me3, K4me3, or POL2).
D. A barplot showing the number of genes with allelic skews in all possible combinations of ChIP marks.
(TIFF)

S2 Fig Allelic assignment of reads, as percentages, for individual ChIPseq experiments. (TIFF)

S3 Fig Number of allelically assigned reads for each chromosome and ChIPseq experiment. (TIFF)

S4 Fig Number of allelically assigned reads for each chromosome for both RNA-seq replicates. (TIFF)

S5 Fig Pairwise composite ChIP enrichment. A. Composite ChIP enrichment for K4me3 and Pol2. Genes are colored according to composite expression as in main text Fig 2.
B. Composite ChIP enrichment for K36me3 and Pol2. Genes are colored according to composite expression as in main text Fig 2.
(TIFF)

S6 Fig Scatter plots of inferred mark densities on paternal (P) *cas* allele for H3K4me3 in TSS-proximal regions vs H3K27me3 in TSS-proximal regions (a) and vs H3K36me3 on gene bodies (b). Color represents allelic levels of expression measured by FPKM values based on RNA-seq.
(TIFF)

S7 Fig Distributions of gene expression and allelic skew of chromatin marks among genes with silent, active, and bivalent chromatin states. **A,** The three regions of K4me3/K27me3 space (Fig 2) correspond to low, intermediate, and high expression levels, respectively (shown as box plots of FPKM values based on RNA-seq).
**B-D,** Distributions of the magnitudes of allelic skew for K27me3 (b), K4me3 (c), and K36me3 (d) among all genes and among three separate categories of chromatin states. Horizontal red dotted lines correspond to 2-fold allelic skew in either direction (log2 ratio of ±1).
(TIFF)

S8 Fig Allelic skew of K4me3 results from specific pairwise combinations of allelic chromatin states in the space of K4me3 and K36me3 densities. For genes with allelic skew of K4me3, maternal and paternal alleles are shown as points in the space of K4me3 densities at TSS-proximal regions and K36me3 densities on gene bodies.
**A,** Scatter plot of K4me3 vs K36me3 densities on maternal (M, *mus*) allele. All autosomal genes are shown as gray points; genes with allelic skew in K4me3 are highlighted in blue. Regions 1, 2, and 3 define maternal alleles with low, medium, and high K4me3 density, respectively.
**B-D,** Scatter plots of K4me3 vs K36me3 densities on paternal (P, *cas*) allele, shown as gray points. Red points in these three plots highlight three separate subgroups of genes shown in **A**: genes whose maternal allele has low, medium, or high K4me3 density (regions 1, 2, and 3 in **A**, marked for the reference by a blue rectangle in each corresponding plot **B-D**). Hue indicates the local density of paternal alleles with similar K4me3/K36me3 densities.
**B,** Among genes with K4me3 skew whose maternal allele has depleted K4me3 (region 1 in **A,** *n* = 76), paternal allele generally has medium to high densities of K4me3. Medium levels of K4me3 largely correspond to bivalent alleles that have depleted levels of K36me3, whereas high levels of K4me3 correspond to active alleles with enriched levels of K36me3 over gene body.
**C,** Among genes with K4me3 skew that have medium (region 2 in **A,** *n* = 273) or high levels of K4me3 on maternal allele (region 3 in **A,** *n* = 371), paternal allele generally also has medium to high densities of K4me3, with a few cases of full depletion of paternal K4me3. The level of K36me3 on these alleles is largely associated with the level of K4me3, with medium levels of K4me3 corresponding to K36me3 depletion and high levels of K4me3 corresponding to K36me3 enrichment.
(TIFF)

S9 Fig Bivalent genes at the composite level are also bivalent at individual alleles. Bivalent promoters at the composite ChIP-seq level are selected based on K4 and K27 occupancy thresholds (green points, n = 1222, in panel A, n = 2780 in panel B). Allelic occupancies for these same genes are shown for both maternal (panels C and D) and paternal (panels D and F) alleles. Non-bivalent genes are colored based on expression values.
(TIFF)

S10 Fig Relationships between the magnitudes of allelic skew of POL2S2 and histone modifications on the same gene. For all genes with sufficient number of allelically assigned reads, ratios of allelic read counts (*mus*:*cas*) are plotted for one mark vs another: **A,** K27me3 vs POL2S2; **B,** K36me3 vs POL2S2. Horizontal and vertical dashed lines mark the 2-fold allelic difference. The majority of genes do not show significant skew in any mark (gray points around origin). Most of the genes with skewed chromatin state (colored points) have a skew in only one mark and no significant skew in the other (purple triangles and magenta squares). However, when both marks are skewed (cyan circles), these skews are anticorrelated for POL2S2 vs repressive mark K27me3 (A) and correlated for POL2S2 vs active mark K36me3 (B).
(TIFF)

S11 Fig Possible pairwise allelic combinations of major types of chromatin states and their impact on allelic expression. All pairwise combinations of three major types of allelic states (silent, bivalent, and active) on maternal (M) and paternal (P) allele are schematically shown as a table. Levels of K4me3 and K27me3, and active expression are indicated for each allele. Only upper-right part of the table is shown; the lower-left part is symmetrical since there is no genome-wide bias with respect to parental genome. Combinations of two alleles with the same major type of chromatin state are shown in white. Combinations of two different types of states are colored; the vast majority of these combinations are observed either between active and silent, or between active and bivalent allele (“observed” combinations, marked in cyan). The combination of bivalent and silent alleles is much less frequent (“unobserved” combination, marked in orange). The skew in allelic expression can result from the combination of active and silent chromatin state, active and bivalent chromatin state, or two quantitatively different chromatin states of active type (c.f. Panel A in Fig 4).
(TIFF)

S12 Fig Percentage of allelically assigned reads for individual RNAseq experiments. (TIFF)

S13 Fig Evaluation of predicted skew in allelic expression based on the skew in densities of ChIP-seq marks. We assessed predictions by linear regression models of expression skew based on the skew of each individual ChIP-seq mark alone and on the combination of all marks. Values of expression skew predicted by these models were classified into “skewed” and “balanced” categories using cutoff values sliding between 0.0 and 6.0. True positive and false positive rates were calculated based on the comparison of predicted categories to observed categories from allele-specific RNA-seq using the cutoffs of 2-fold skew and FDR of 0.05. The resulting receiver operating characteristic (ROC) curves were plotted for the predictions based on the skew of K4me3 (black), K36me3 (red), Pol2S2 (green), K27me3 (blue), or all marks together (magenta).
(TIFF)

S14 Fig Comparison of mus:cas expression skew with differential expression between inbred mus musculus and mus castaneus mice. A. Comparison of gene expression between MEFs of inbred homozygous mice by RNAseq. Only genes with total reads > 15 between both parents are shown. Autosomal genes are shown in black, X-linked genes are shown in orange.
B. Hybrid mouse expression skew compared with differential expression, computed as expression skew, in homozygous MEFs. Genes skewed as a result of genetic differences, sequence independent mechanisms, or other factors are highlighted in magenta, red, and blue, respectively.
(TIFF)

S1 Table Proportion of expression skew variance explained by the skew of each mark individually (first row) and the increments gained by the inclusion of each additional mark in a multivariate model, in the order K4-K36-Pol2S2-K27 (second row). The proportion explained by a combined model with all marks is 0.57.
(XLSX)

S2 Table Excel file with tables of genes with significant allelic skew detected in K27me3, K36me3, K4me3, POL2S2, and POL2S5 ChIP-seq coverage. In each table, gene name, chromosomal coordinates, strand, total (composite) number of mapped reads, numbers of allele-specific reads for maternal (*mus*) and paternal (*cas*) alleles, allelic skew as the logarithm of *mus*:*cas* ratio, and Benjamini-Hochberg FDR are indicated.
(XLSX)

S3 Table Excel file with tables of genes with 5 or more allele-specific reads assigned to at least one allele. In each table, gene name, chromosomal coordinates, strand, total (composite) number of mapped reads, numbers of allele-specific reads for maternal (*mus*) and paternal (*cas*) alleles, allelic skew as the logarithm of *mus*:*cas* ratio, and Benjamini-Hochberg FDR are indicated.
(XLSX)

S4 Table Spearman correlation coefficients of allelic skew for all pairs of surveyed ChIP-Seq marks (K27me3, K36me3, K4me3, POL2S2, and POL2S5). Correlation was calculated based on genes where both compared marks have significant allelic skew.
(XLSX)

S5 Table Excel file with the table of genes with significant allelic skew in expression based on RNA-seq. Gene name, number of fragments per kilobase per million (FPKM) based on all reads, counts of allele-specific reads assigned to maternal (*mus*) and paternal (*cas*) alleles in two replicates, log-ratio of average allelic read counts, and false discovery rate (FDR) of the skew are indicated.
(XLSX)
