# Supplementary figures and images for: Genome-wide identification of autosomal genes with allelic imbalance of chromatin state
Source: PLoS One. 2017 Aug 10;12(8):e0182568. doi: 10.1371/journal.pone.0182568 (PMC5552117; doi:10.1371/journal.pone.0182568)

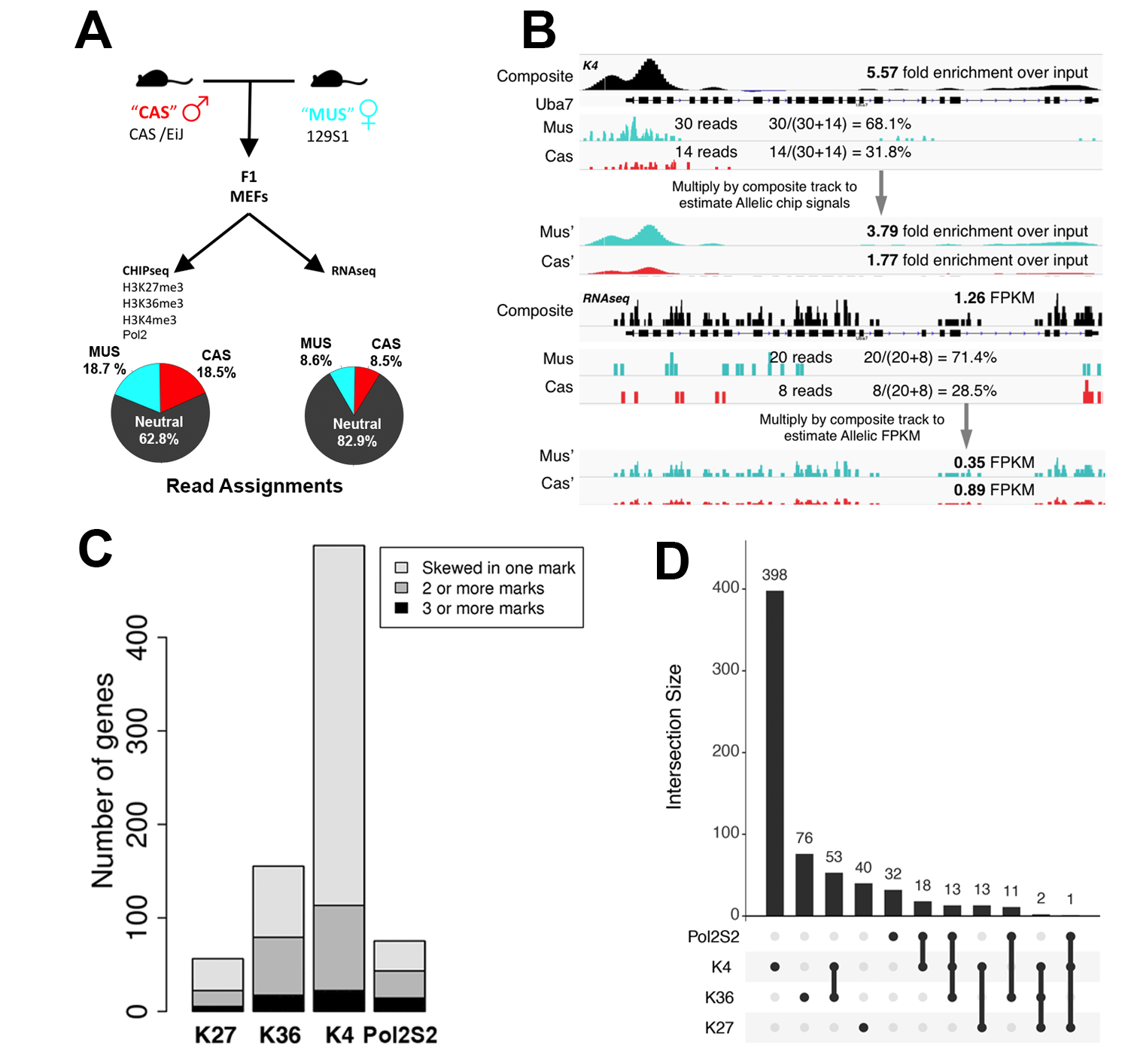

Supplement: S1 Fig — A. ChIP-seq and RNA-seq reads that overlap mus/cas SNPs (ChIP-seq approx. 37%, RNA-seq approx. 17%, of total reads) can be assigned uniquely to one parental allele: mus, maternal; cas, paternal. B. A methodology schematic showing the calculation of allelic densities and expression for ChIP seq and RNA seq reads, respectively. C. A fraction of genes showed simultaneous allelic skew in two or three marks. The barplot shows the numbers of genes with simultaneous skew in 2 or 3 marks as fractions of genes with skew in each individual mark (K27me3, K36me3, K4me3, or POL2). D. A barplot showing the number of genes with allelic skews in all possible combinations of ChIP marks. (TIFF) [file pone.0182568.s001.tiff]

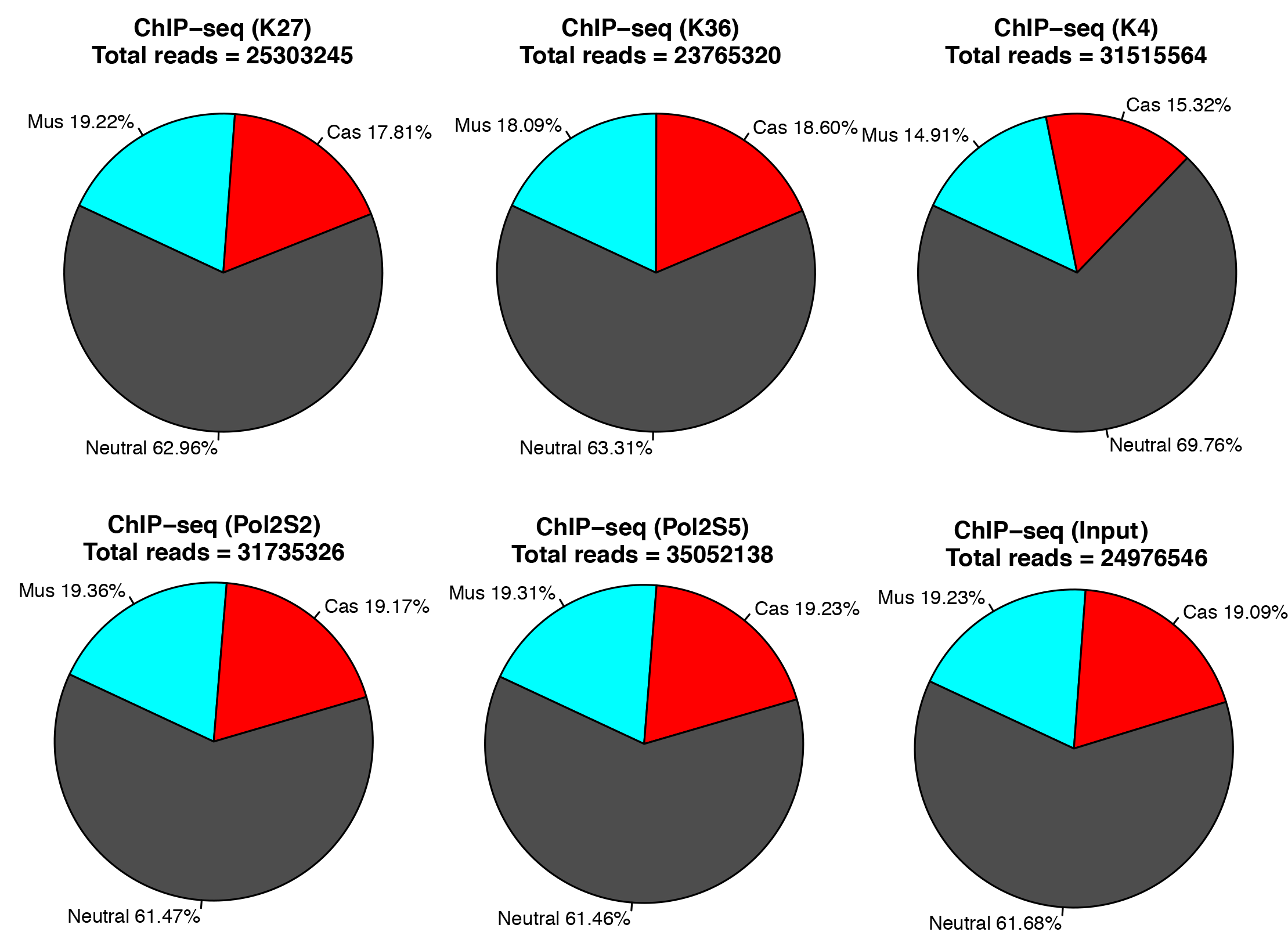

Supplement: S2 Fig — (TIFF) [file pone.0182568.s002.tiff]

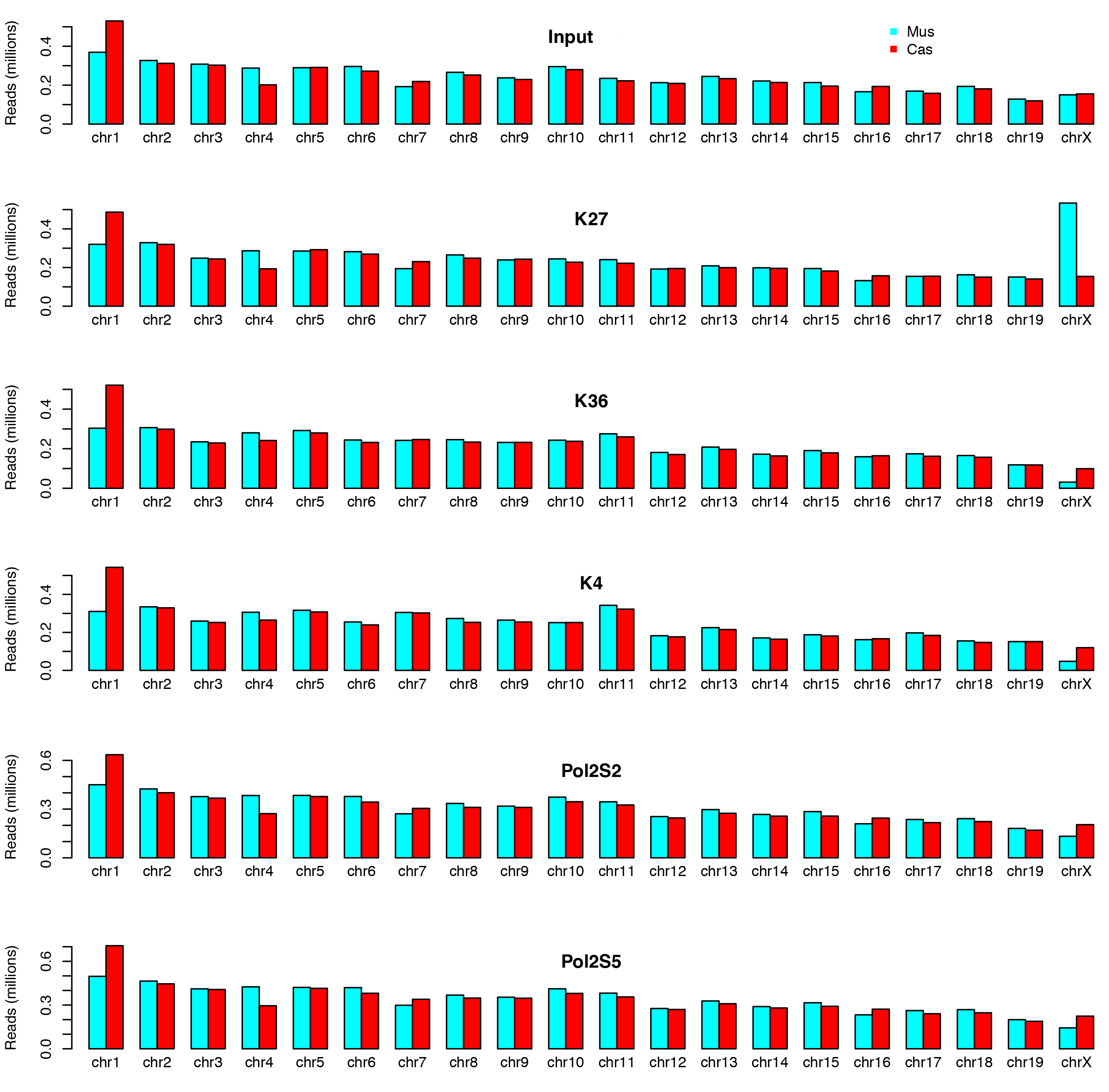

Supplement: S3 Fig — (TIFF) [file pone.0182568.s003.tiff]

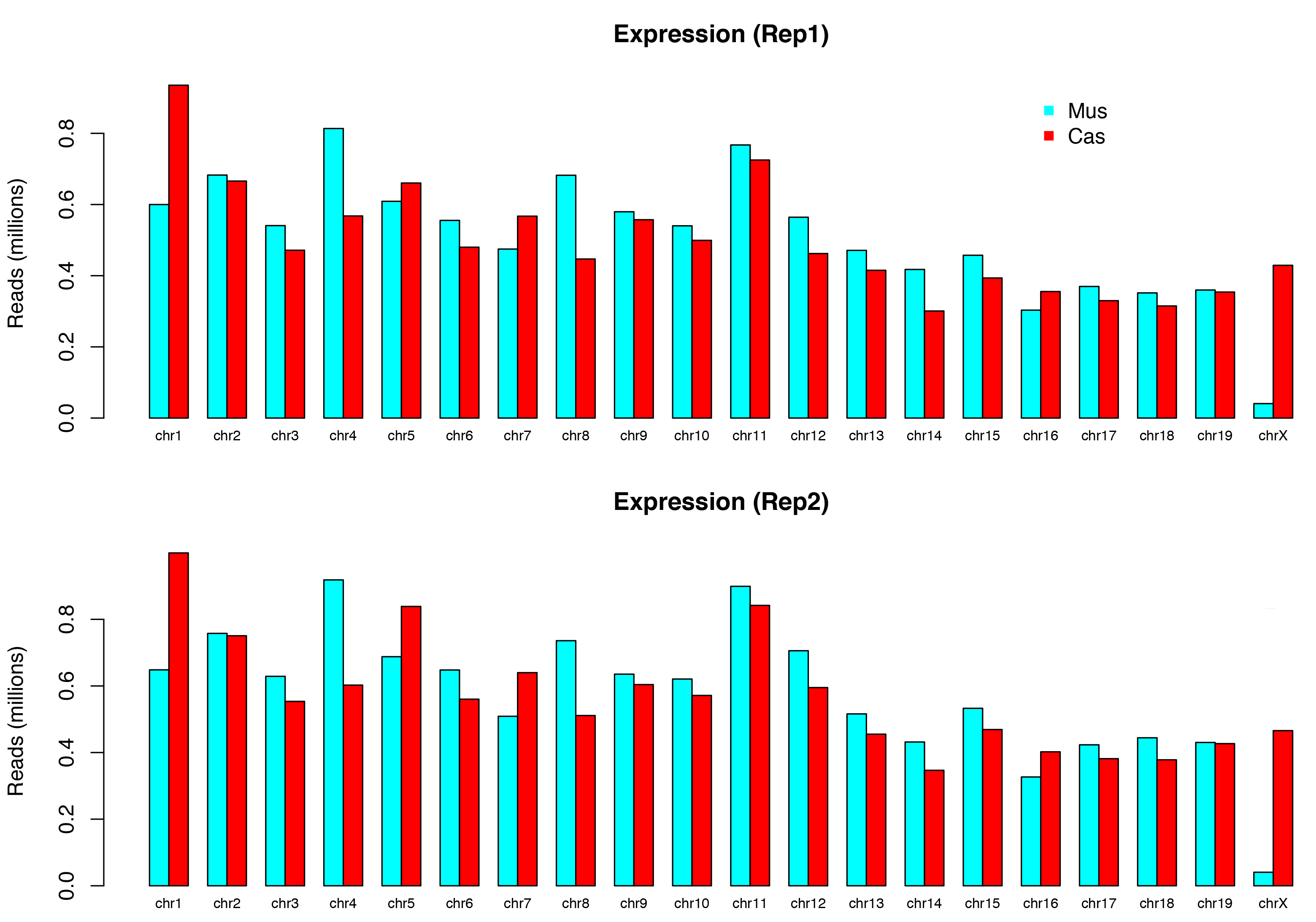

Supplement: S4 Fig — (TIFF) [file pone.0182568.s004.tiff]

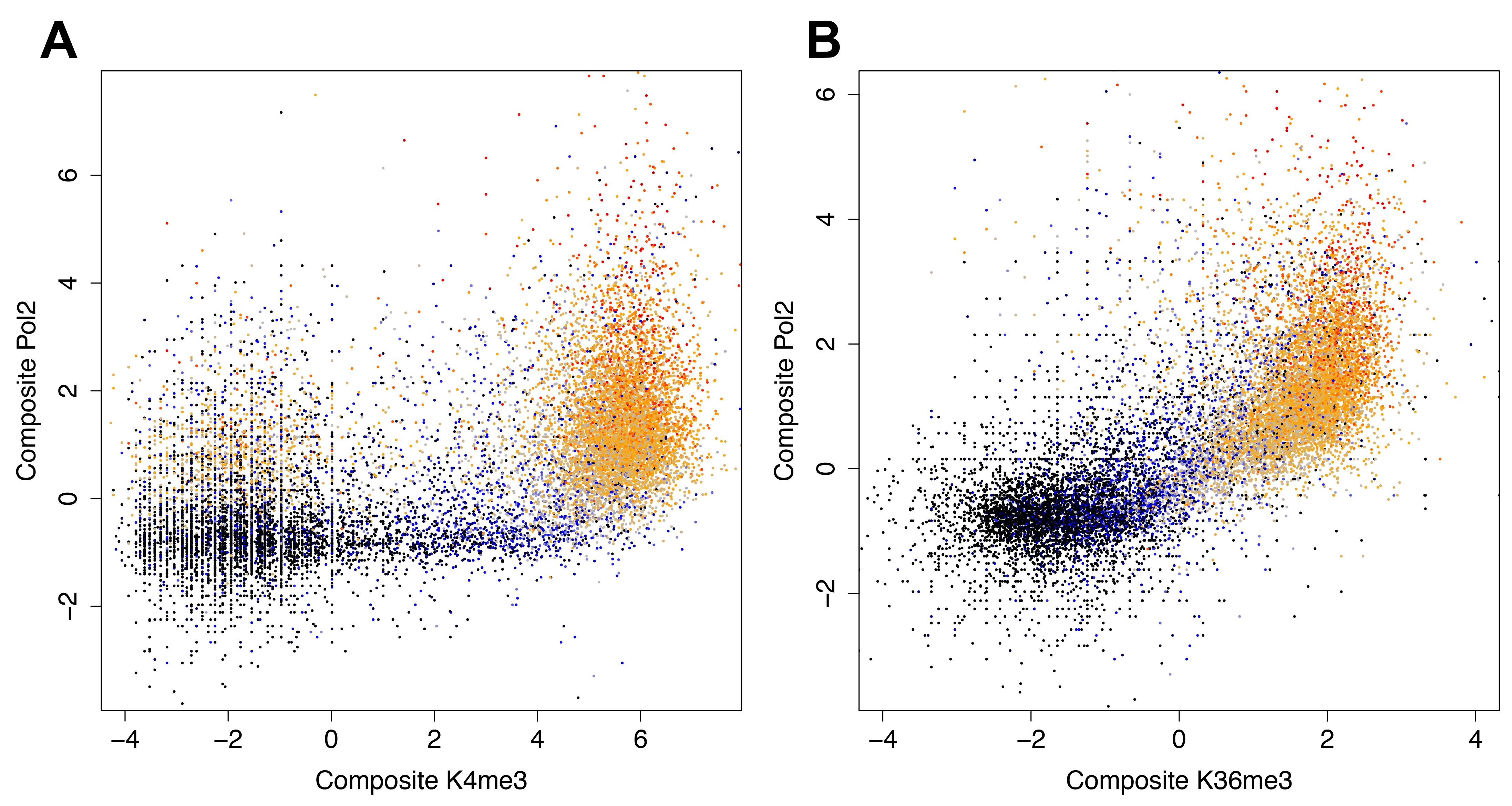

Supplement: S5 Fig — A. Composite ChIP enrichment for K4me3 and Pol2. Genes are colored according to composite expression as in main text Fig 2. B. Composite ChIP enrichment for K36me3 and Pol2. Genes are colored according to composite expression as in main text Fig 2. (TIFF) [file pone.0182568.s005.tiff]

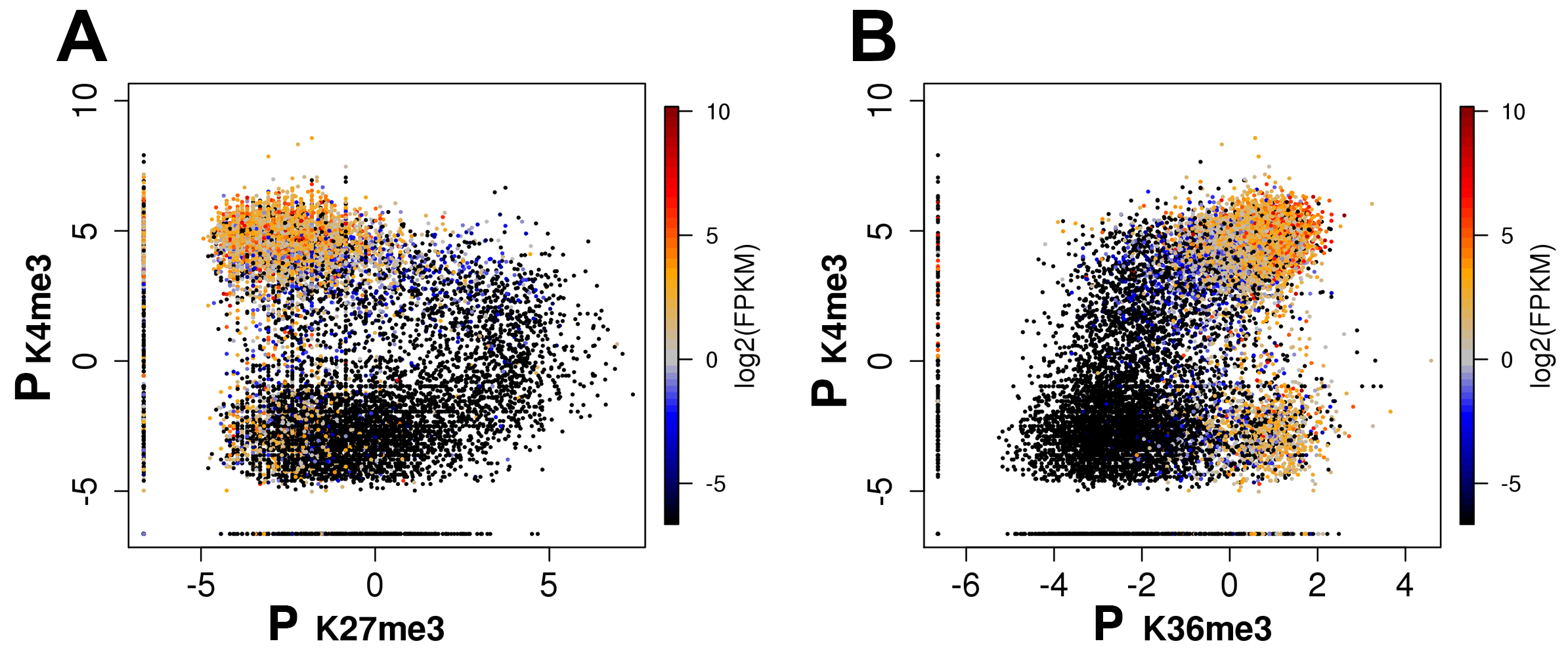

Supplement: S6 Fig — Scatter plots of inferred mark densities on paternal (P) cas allele for H3K4me3 in TSS-proximal regions vs H3K27me3 in TSS-proximal regions (a) and vs H3K36me3 on gene bodies (b). Color represents allelic levels of expression measured by FPKM values based on RNA-seq. (TIFF) [file pone.0182568.s006.tiff]

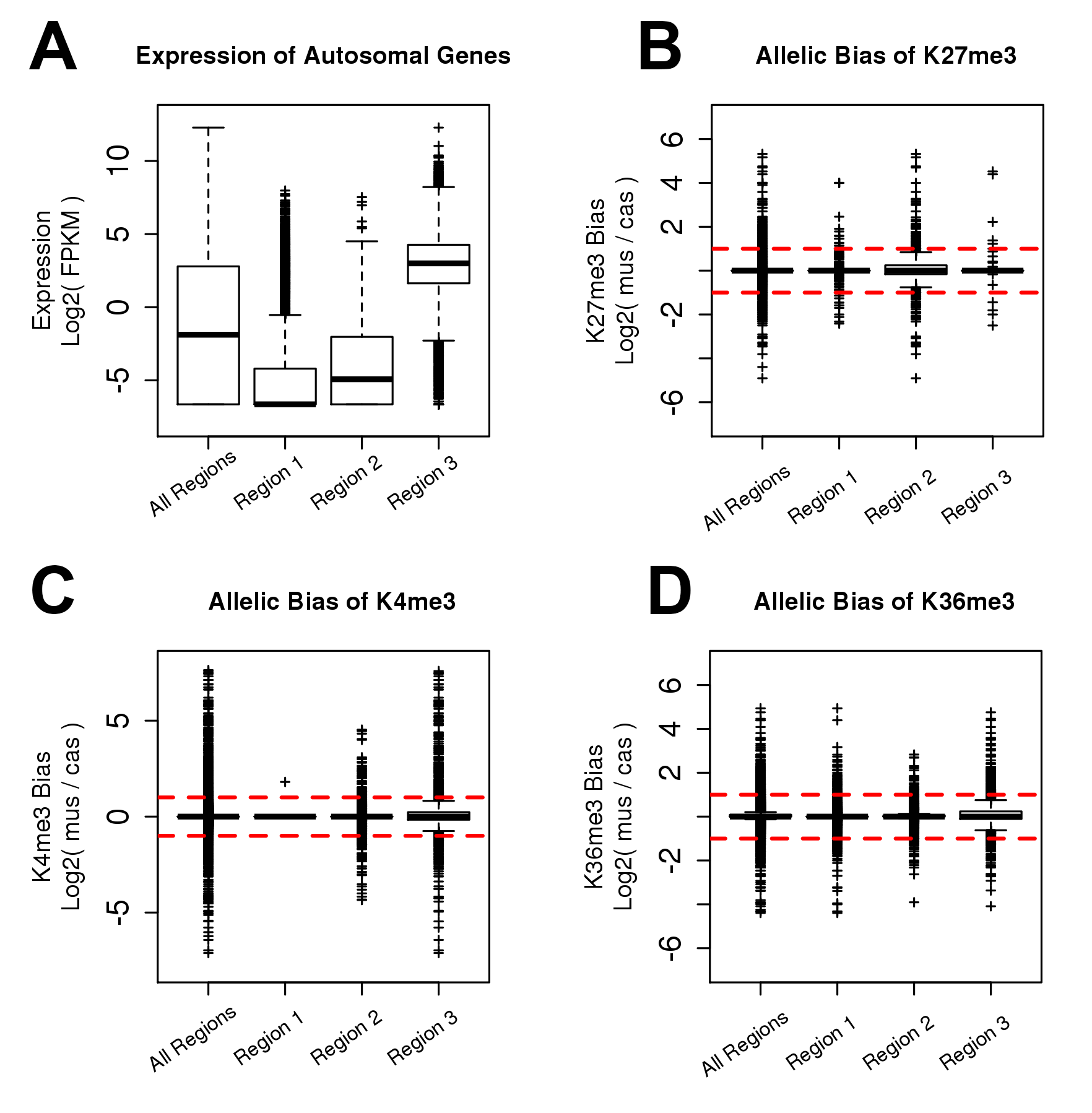

Supplement: S7 Fig — A, The three regions of K4me3/K27me3 space (Fig 2) correspond to low, intermediate, and high expression levels, respectively (shown as box plots of FPKM values based on RNA-seq). B-D, Distributions of the magnitudes of allelic skew for K27me3 (b), K4me3 (c), and K36me3 (d) among all genes and among three separate categories of chromatin states. Horizontal red dotted lines correspond to 2-fold allelic skew in either direction (log2 ratio of ±1). (TIFF) [file pone.0182568.s007.tiff]

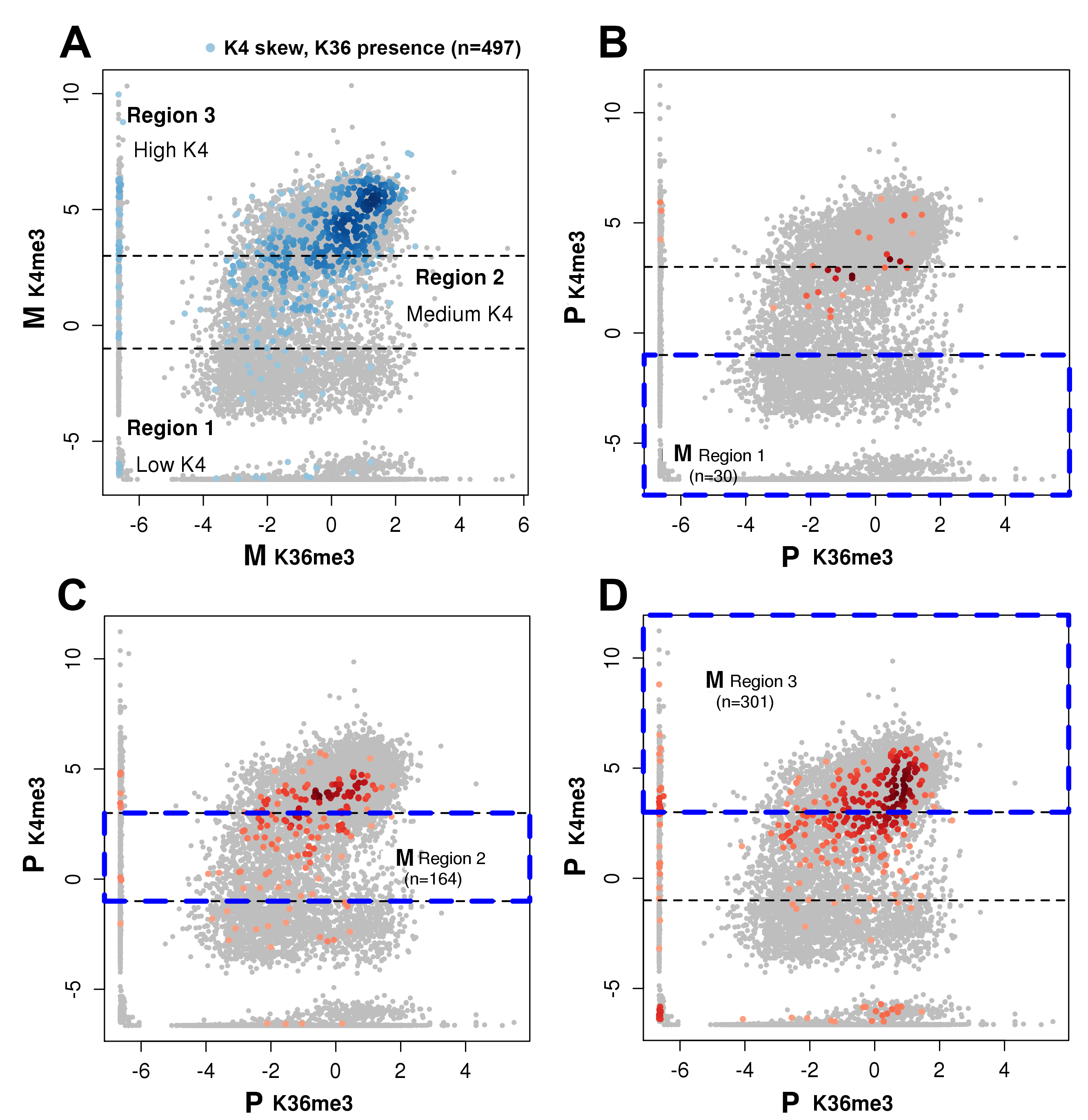

Supplement: S8 Fig — For genes with allelic skew of K4me3, maternal and paternal alleles are shown as points in the space of K4me3 densities at TSS-proximal regions and K36me3 densities on gene bodies. A, Scatter plot of K4me3 vs K36me3 densities on maternal (M, mus) allele. All autosomal genes are shown as gray points; genes with allelic skew in K4me3 are highlighted in blue. Regions 1, 2, and 3 define maternal alleles with low, medium, and high K4me3 density, respectively. B-D, Scatter plots of K4me3 vs K36me3 densities on paternal (P, cas) allele, shown as gray points. Red points in these three plots highlight three separate subgroups of genes shown in A: genes whose maternal allele has low, medium, or high K4me3 density (regions 1, 2, and 3 in A, marked for the reference by a blue rectangle in each corresponding plot B-D). Hue indicates the local density of paternal alleles with similar K4me3/K36me3 densities. B, Among genes with K4me3 skew whose maternal allele has depleted K4me3 (region 1 in A, n = 76), paternal allele generally has medium to high densities of K4me3. Medium levels of K4me3 largely correspond to bivalent alleles that have depleted levels of K36me3, whereas high levels of K4me3 correspond to active alleles with enriched levels of K36me3 over gene body. C, Among genes with K4me3 skew that have medium (region 2 in A, n = 273) or high levels of K4me3 on maternal allele (region 3 in A, n = 371), paternal allele generally also has medium to high densities of K4me3, with a few cases of full depletion of paternal K4me3. The level of K36me3 on these alleles is largely associated with the level of K4me3, with medium levels of K4me3 corresponding to K36me3 depletion and high levels of K4me3 corresponding to K36me3 enrichment. (TIFF) [file pone.0182568.s008.tiff]

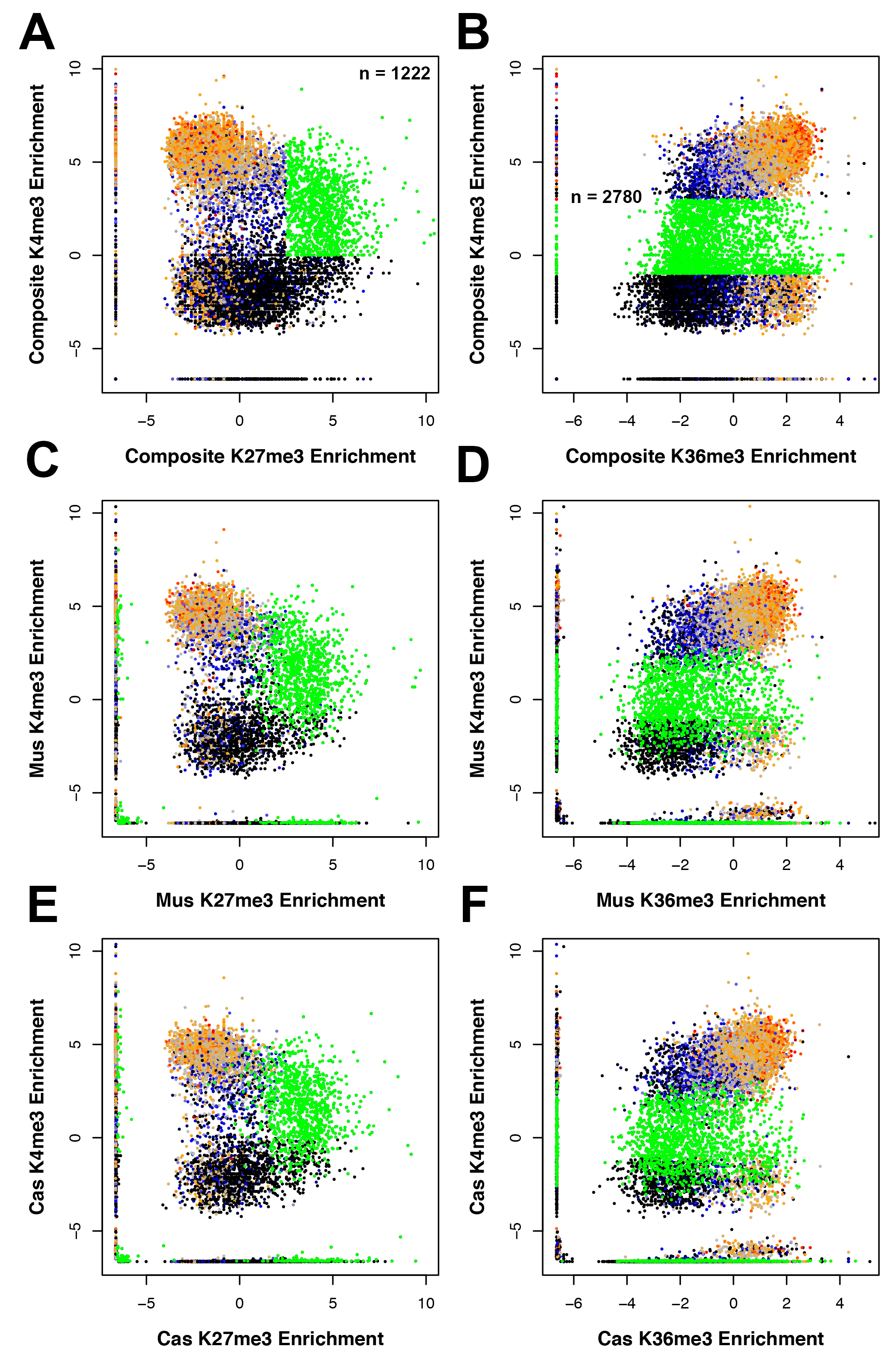

Supplement: S9 Fig — Bivalent promoters at the composite ChIP-seq level are selected based on K4 and K27 occupancy thresholds (green points, n = 1222, in panel A, n = 2780 in panel B). Allelic occupancies for these same genes are shown for both maternal (panels C and D) and paternal (panels D and F) alleles. Non-bivalent genes are colored based on expression values. (TIFF) [file pone.0182568.s009.tiff]

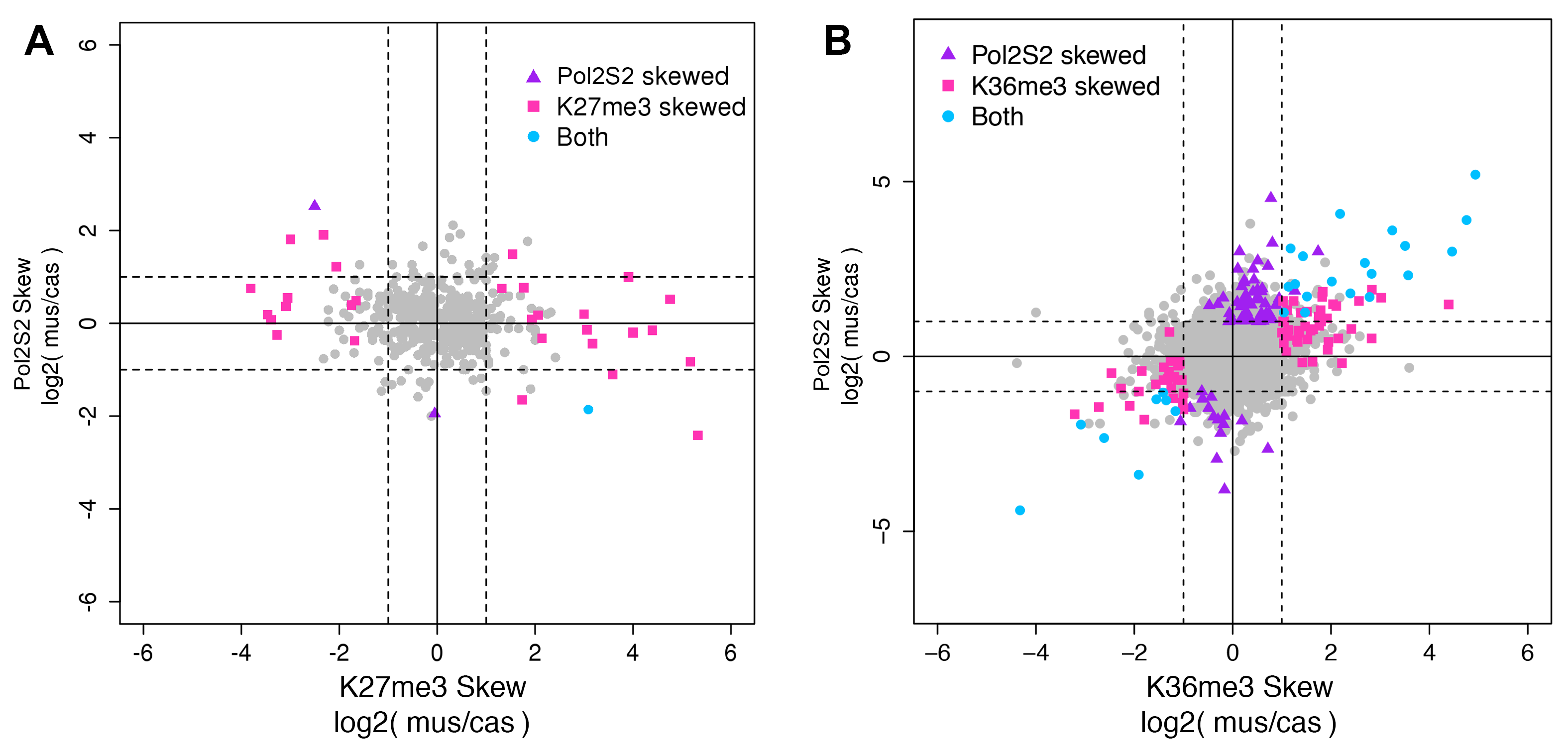

Supplement: S10 Fig — For all genes with sufficient number of allelically assigned reads, ratios of allelic read counts (mus:cas) are plotted for one mark vs another: A, K27me3 vs POL2S2; B, K36me3 vs POL2S2. Horizontal and vertical dashed lines mark the 2-fold allelic difference. The majority of genes do not show significant skew in any mark (gray points around origin). Most of the genes with skewed chromatin state (colored points) have a skew in only one mark and no significant skew in the other (purple triangles and magenta squares). However, when both marks are skewed (cyan circles), these skews are anticorrelated for POL2S2 vs repressive mark K27me3 (A) and correlated for POL2S2 vs active mark K36me3 (B). (TIFF) [file pone.0182568.s010.tiff]

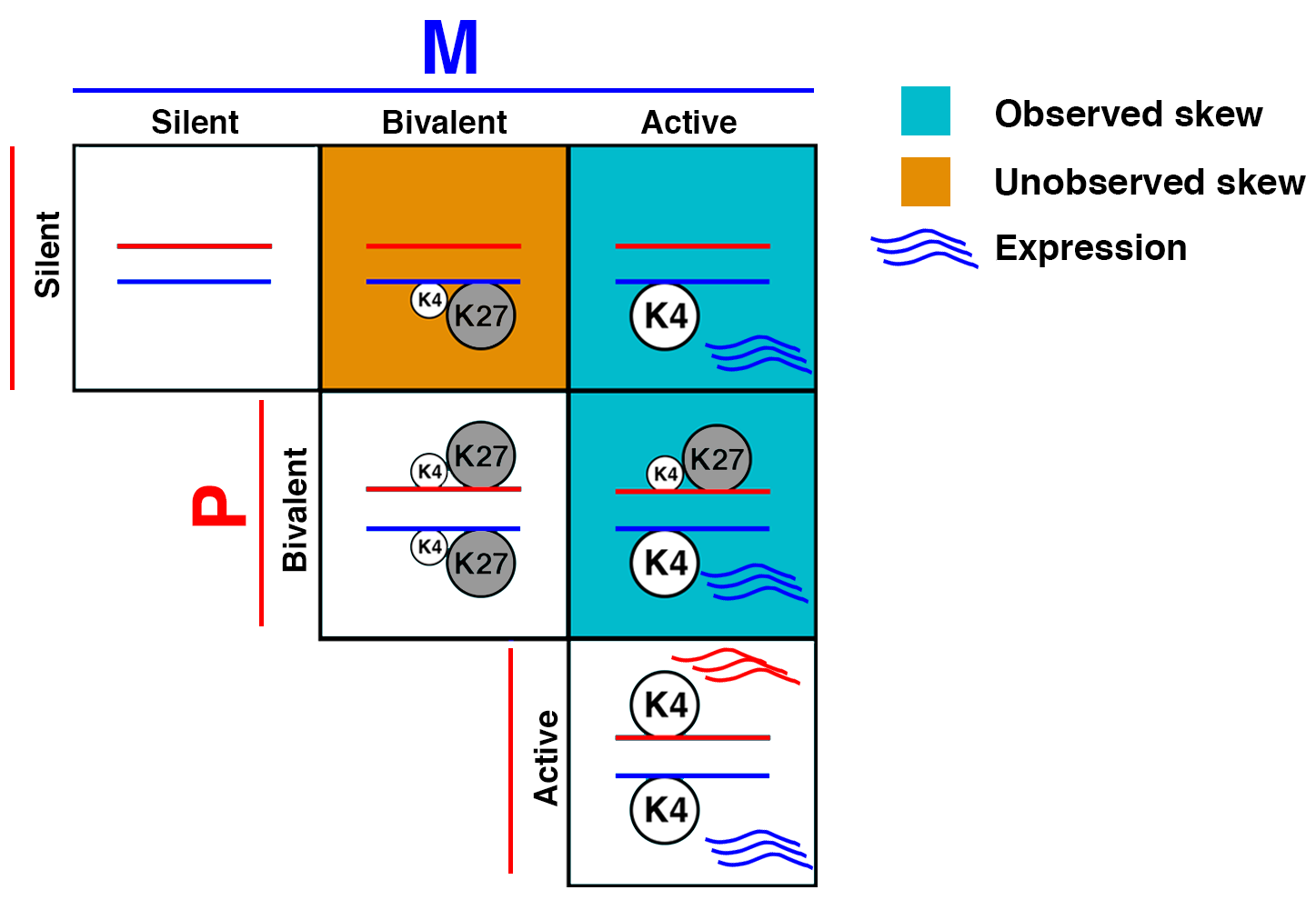

Supplement: S11 Fig — All pairwise combinations of three major types of allelic states (silent, bivalent, and active) on maternal (M) and paternal (P) allele are schematically shown as a table. Levels of K4me3 and K27me3, and active expression are indicated for each allele. Only upper-right part of the table is shown; the lower-left part is symmetrical since there is no genome-wide bias with respect to parental genome. Combinations of two alleles with the same major type of chromatin state are shown in white. Combinations of two different types of states are colored; the vast majority of these combinations are observed either between active and silent, or between active and bivalent allele (“observed” combinations, marked in cyan). The combination of bivalent and silent alleles is much less frequent (“unobserved” combination, marked in orange). The skew in allelic expression can result from the combination of active and silent chromatin state, active and bivalent chromatin state, or two quantitatively different chromatin states of active type (c.f. Panel A in Fig 4). (TIFF) [file pone.0182568.s011.tiff]

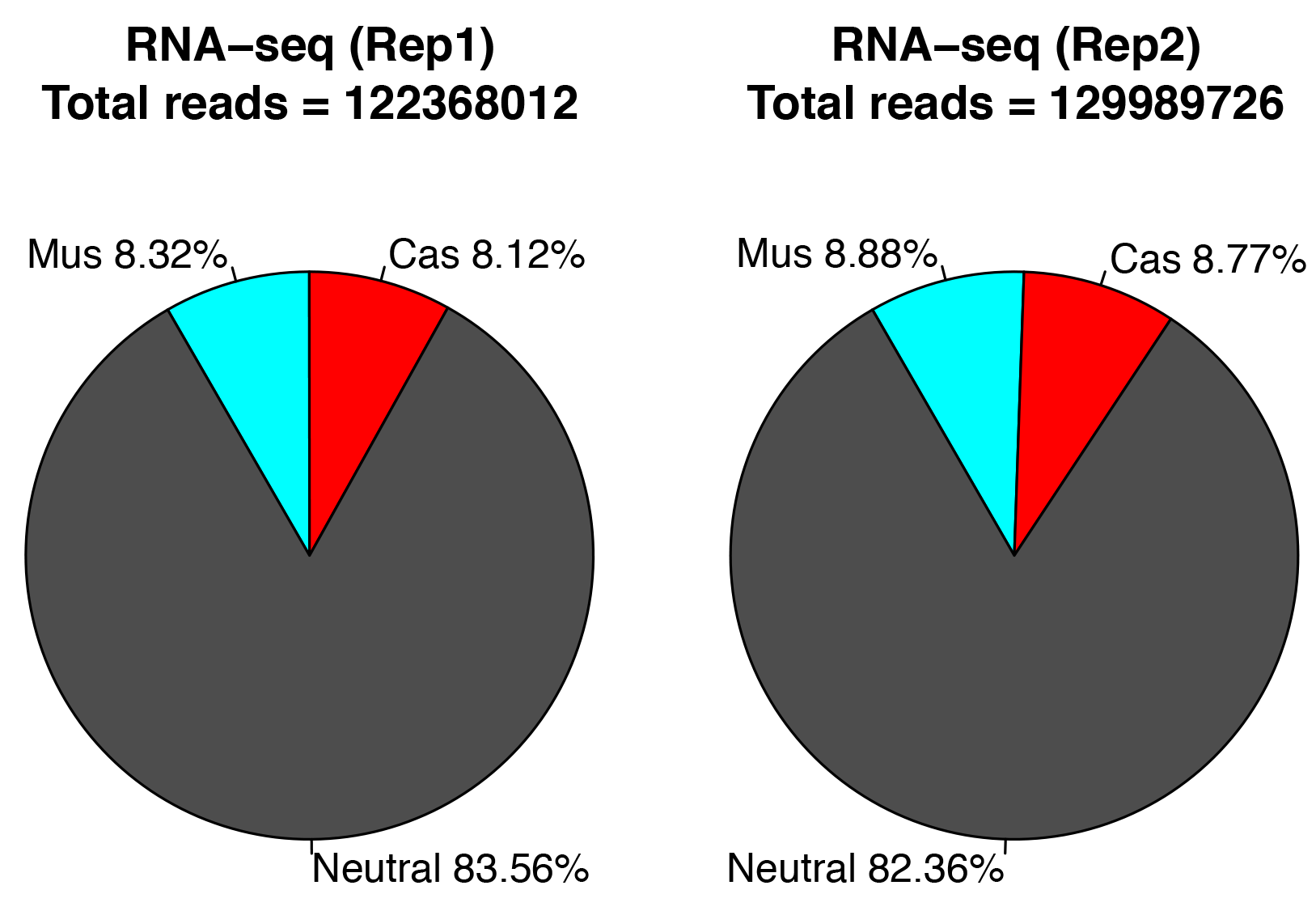

Supplement: S12 Fig — (TIFF) [file pone.0182568.s012.tiff]

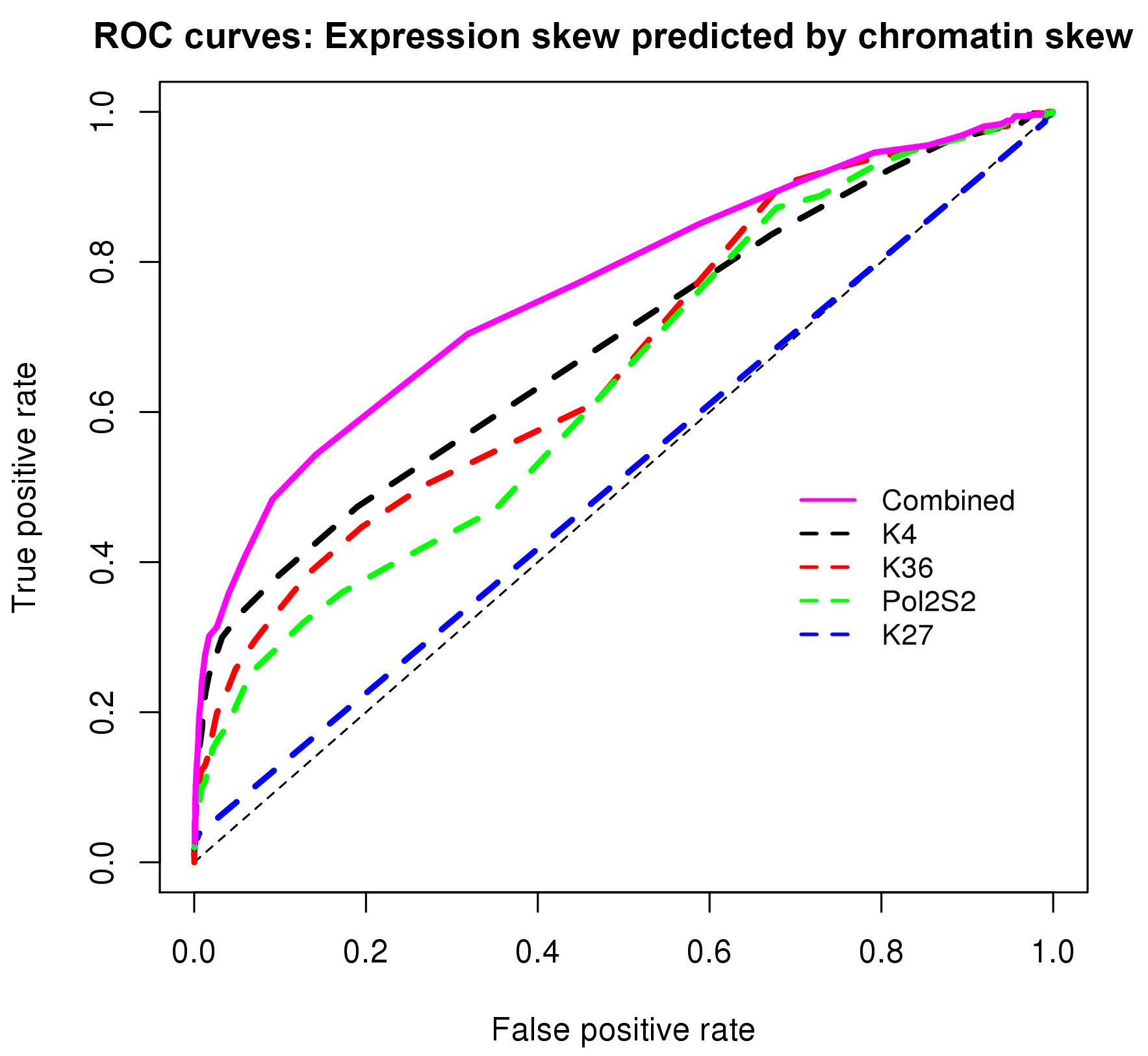

Supplement: S13 Fig — We assessed predictions by linear regression models of expression skew based on the skew of each individual ChIP-seq mark alone and on the combination of all marks. Values of expression skew predicted by these models were classified into “skewed” and “balanced” categories using cutoff values sliding between 0.0 and 6.0. True positive and false positive rates were calculated based on the comparison of predicted categories to observed categories from allele-specific RNA-seq using the cutoffs of 2-fold skew and FDR of 0.05. The resulting receiver operating characteristic (ROC) curves were plotted for the predictions based on the skew of K4me3 (black), K36me3 (red), Pol2S2 (green), K27me3 (blue), or all marks together (magenta). (TIFF) [file pone.0182568.s013.tiff]

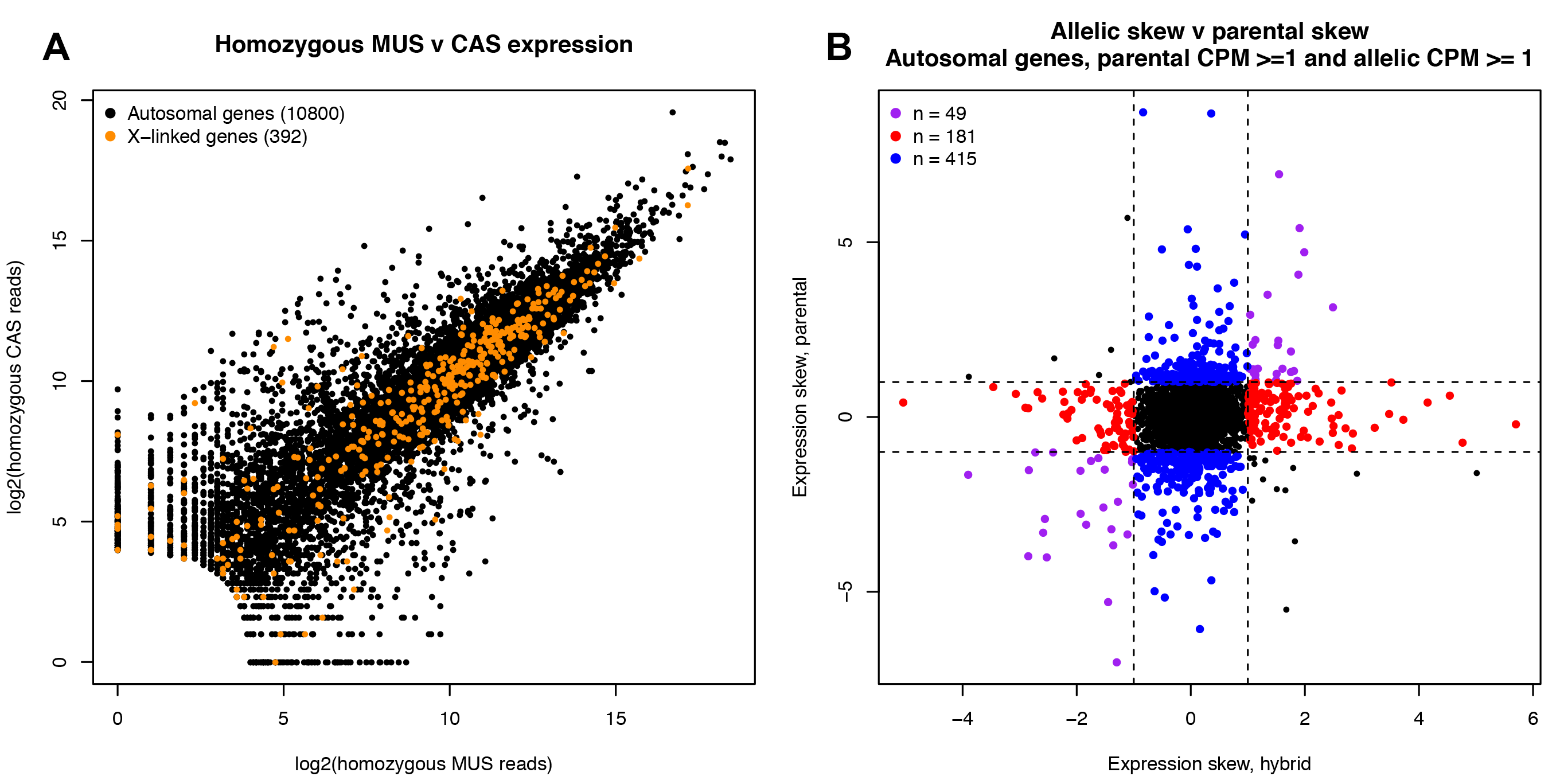

Supplement: S14 Fig — A. Comparison of gene expression between MEFs of inbred homozygous mice by RNAseq. Only genes with total reads > 15 between both parents are shown. Autosomal genes are shown in black, X-linked genes are shown in orange. B. Hybrid mouse expression skew compared with differential expression, computed as expression skew, in homozygous MEFs. Genes skewed as a result of genetic differences, sequence independent mechanisms, or other factors are highlighted in magenta, red, and blue, respectively. (TIFF) [file pone.0182568.s014.tiff]
